# Administration of Gas6 attenuates lung fibrosis via inhibition of the epithelial-mesenchymal transition and fibroblast activation

**DOI:** 10.1007/s10565-024-09858-5

**Published:** 2024-04-05

**Authors:** Ye-Ji Lee, Minsuk Kim, Hee-Sun Kim, Jihee Lee Kang

**Affiliations:** 1 Department of Physiology, College of Medicine, Ewha Womans University, 25 Magokdong-Ro 2-Gil, Gangseo-Gu, Seoul, 07804 Korea; 2 Department of Pharmacology, College of Medicine, Ewha Womans University, 25 Magokdong-Ro 2-Gil, Gangseo-Gu, Seoul, 07804 Korea; 3 Department of Molecular Medicine, College of Medicine, Ewha Womans University, 25 Magokdong-Ro 2-Gil, Gangseo-Gu, Seoul, 07804 Korea; 4 Inflammation-Cancer Microenvironment Research Center, College of Medicine, Ewha Womans University, 25 Magokdong-Ro 2-Gil, Gangseo-Gu, Seoul, 07804 Korea

**Keywords:** Gas6, Axl, EMT, Fibroblast activation, Pulmonary fibrosis

## Abstract

The epithelial-mesenchymal transition (EMT) and fibroblast activation are major events in idiopathic pulmonary fibrosis pathogenesis. Here, we investigated whether growth arrest-specific protein 6 (Gas6) plays a protective role in lung fibrosis via suppression of the EMT and fibroblast activation. rGas6 administration inhibited the EMT in isolated mouse ATII cells 14 days post-BLM treatment based on morphologic cellular alterations, changes in mRNA and protein expression profiles of EMT markers, and induction of EMT-activating transcription factors. BLM-induced increases in gene expression of fibroblast activation-related markers and the invasive capacity of primary lung fibroblasts in primary lung fibroblasts were reversed by rGas6 administration. Furthermore, the hydroxyproline content and collagen accumulation in interstitial areas with damaged alveolar structures in lung tissue were reduced by rGas6 administration. Targeting Gas6/Axl signaling events with specific inhibitors of Axl (BGB324), COX-2 (NS-398), EP1/EP2 receptor (AH-6809), or PGD2 DP2 receptor (BAY-u3405) reversed the inhibitory effects of rGas6 on EMT and fibroblast activation. Finally, we confirmed the antifibrotic effects of Gas6 using Gas6^−/−^ mice. Therefore, Gas6/Axl signaling events play a potential role in inhibition of EMT process and fibroblast activation via COX-2-derived PGE_2_ and PGD_2_ production, ultimately preventing the development of pulmonary fibrosis.

**Supplementary Information:**

The online version contains supplementary material available at 10.1007/s10565-024-09858-5.

## Background

Fibrosis is a defining feature of Idiopathic Pulmonary Fibrosis (IPF), a lung disorder that advances progressively. IPF represents an irreversible progression marked by damage to alveolar epithelial cells, accumulation of fibroblasts, and their subsequent differentiation into myofibroblasts, which increases extracellular matrix (ECM) accumulation and leads to irreversible distortion of the lung parenchyma. This disease is generally a fatal disorder of unknown etiology, and the anticipated average survival spans from 2 to 5 years after the moment of diagnosis (Phan et al. 2021). Currently sanctioned treatments decelerate the advancement of IPF but do not provide a cure, highlighting the need for new therapeutic approaches (Somogyi et al. 2019).

Apoptosis, cellular senescence, epithelial-mesenchymal transition (EMT), endothelial transition to a mesenchymal state, and migration of epithelial cells have been identified as pivotal processes contributing to the tissue remodeling associated with IPF (Somogyi et al. 2019). Initially, it was noted that myofibroblasts in the local tissue served as the primary contributors to ECM components in response to injury (Plikus et al. 2021). However, it is currently thought that activated fibroblasts and myofibroblasts could arise from other cells, including bone marrow-derived circulating fibrocytes (Quan et al. 2006), endothelial cells (Hashimoto et al. 2010), microvascular pericytes (Lin et al. 2008), and alveolar epithelial cells (Kasai et al. 2005). Indeed, EMT takes place in normal lung development and regeneration after damage. According to the most widely accepted pathogenic mechanisms of IPF, the dysregulated EMT process is recognized as a significant phenomenon in the progression of IPF (Salton et al. 2019). Recent discoveries indicate that alveolar epithelial type II (ATII) cells undergoing EMT are essential for the development of a pro-fibrotic millieu through the release of signaling molecules, thereby initiating the activation of adjacent fibroblasts (Hill et al. 2019). However, the EMT has not yet been fully explored as a possible therapeutic target for fibrosis.

Gas6, also known as growth arrest-specific protein 6, is a member of the vitamin K-dependent protein family found in plasma, sharing structural characteristics. Gas6 engages with its receptors, namely Tyro3, Axl, and Mer (TAM) (Lew et al. 2014). Binding of ligands to the structures resembling immunoglobulins of TAM receptors initiates intracellular tyrosine autophosphorylation, leading to signal transduction through distinct pathways. TAM signaling is crucial in modulating the innate immunity. Previous studies indicate that Gas6/Mer or Axl signaling provides a protective effect in mouse models of multi-organ failure syndrome and acute lung injury, (Salmi et al. 2021; Kim et al. 2021; Peng et al. 2019), and acute liver injury (Zagórska et al. 2020). In contrast, genetic deficiency of Gas6 or Mer in mice provides protection against pulmonary inflammation and fibrosis triggered by silica exposure. (Li et al. 2019). A recent investigation also illustrates that small molecular inhibitors targeting TAM receptors mitigated the properties of fibroblasts isolated from IPF patients, including synthetic and migratory characteristics (Espindola et al. 2018). Conversely, the administration of protein S, an alternate ligand binding TAM receptor, has been illustrated to hinder pulmonary fibrosis in mice exposed to bleomycin (BLM) and prevent apoptosis in alveolar epithelial cells (Urawa et al. 2016). In comparison, our earlier research has illustrated that Gas6 plays a role in maintaining homeostasis of alveolar epithelial cells by regulating proliferation and tissue repair processes (Lee et al. 2014; Park et al. 2012). Furthermore, data from our previous in vitro study suggest that Gas6 signaling events may reprogram lung epithelial cells to prevent EMT by the synthesis of prostaglandin E_2_ (PGE_2_) and PGD_2_ derived from COX-2, and their receptors (Jung et al. 2019). However, whether Gas6 suppresses alveolar epithelial type II (ATII) cells in the process of EMT and inhibits fibroblast activation to prevent lung fibrosis in vivo is still unknown.

In this study, we explored if injecting murine recombinant Gas6 (rGas6) suppresses the EMT process and apoptosis in primary ATII cells and concomitantly inhibits fibroblast activation to protect against the progression of pulmonary fibrosis in mice treated with BLM. Our analysis involved examining whether the administration of rGas6 could augment the activation of Axl or Mer signaling pathways, specifically focusing on the pathways involving COX-2 and the synthesis of PGE_2_ and PGD_2_. To elucidate the functional relevance of Gas6/Axl signaling in mediating the suppressive impacts of rGas6 on EMT process and the activation of fibroblast following BLM treatment, we utilized specific inhibitors, including the Axl selective inhibitor BGB324, the NS-398 compound, serving as a COX-2 inhibitor, and the receptor antagonists targeting PGE_2_ and PGD_2_, such as EP2/EP4 (AH-6809) and DP2 (BAY-u3405). Furthermore, using Gas6^−/−^ mice, we confirmed that endogenous Gas6 contributes to the prevention of the EMT and profibrotic response in BLM-induced fibrosis.

## Methods

### Reagents

The suppliers of paraformaldehyde and BLM were Sigma-Aldrich (St Louis, MO, USA). Mouse rGas6 (986-GS) was acquired from R&D Systems (Minneapolis, MN, USA). We bought BGB324 from MedchemExpress in Carlsbad, California, USA. Cayman Chemical (Ann Arbor, MI, USA) was the source of AH-6809, BAY-u3405, and NS-398. Table S1 lists the antibodies used in immunofluorescence and Western blotting.

### Animal protocols

For every test, 20–25 g male C57BL/6 mice were utilized. Their specific pathogen-free status was confirmed after they were acquired from Orient Bio (Sungnam, Korea). *Gas6* ^−/−^ mice have been previously described (Tirado-Gonzalez et al. 2021). C57BL/6 *Gas6*^−/−^ mice (Gas6^tm1.1(KOMP)Vlcg^) were acquired from the Knock Out Mouse Project (KOMP) Repository. The knockout allele’s scheme and thorough description are available at http://www.mousephenotype.org/data/alleles/MGI:95660/tm1.1(KOMP)Vlcg. To verify that Gas6 was absent, Western blot, real-time PCR, and immunocytochemistry assays were performed. The Ewha Medical Research Institute's Animal Care Committee approved the experimental protocol (ESM 17–0369). All procedures involving mice were guided by the National Institutes of Health Guide for the Care and Use of Laboratory Animals. Using mouse pharyngeal instillation, BLM (5 U/kg body weight in 30 μl sterile saline) in a test solution was delivered (Lee et al. 2017; Yoon et al. 2013). One day prior to BLM treatment, rGas6 (50 µg/kg) was injected intraperitoneally (i.p.) and then every two days following that (Peng et al. 2019; Kim et al. 2021). The mice were put to death on day 14 or day 21 after receiving BLM treatment.

For experiments employing the inhibitors, BGB324 (5 mg/kg, intraorally, i.o.) (Espindola et al. 2018), NS-398 (5 mg/kg, i.o.), AH-6809 (5 mg/kg, i.p.) (Yoon et al. 2016), or BAY-u3405 (30 mg/kg, i.o.) (Stelling et al. 2020) was administered at the same time as rGas6 1 day before BLM treatment. The inhibitor was administered once daily (AH-6809) or every two days (BGB324, NS-398, and BAY-u3405) following the first dosage, and mice were sacrificed 14 days after BLM treatment.

### Bronchoalveolar lavage (BAL), cell counts, and lung tissue

BAL was conducted according to previously described methods (Lee et al. 2017; Yoon et al. 2013). After BAL samples were centrifuged at 500 × g for 5 min at 4 °C, cell pellets were cleaned and reconstituted in phosphate-buffered medium. The number of cells was counted using an electronic Coulter Counter equipped with a cell-sizing analyzer (Coulter Model ZBI with a channelizer 256; Coulter Electronics, Bedfordshire, UK). Alveolar macrophages were distinguished by the distinctive diameters of their cells. The lungs were taken out after BAL, and preserved using liquid nitrogen freezing.

### Isolation of ATII cells

Using previously published techniques, primary ATII cells were extracted from mice (Bortnick et al. 2003; Lee et al. 2017). In summary, the lungs were perfused with a solution of 0.9% saline by injecting it into the pulmonary artery until the blood was extracted. After the lungs were rinsed with 1 ml saline, 100 units of dispase were injected, and the lungs were incubated for 45 min at room temperature. The lung tissue was then physically detached from the major bronchi and placed in a Petri dish that was kept at 37 °C for 10 min with Dulbecco's modified Eagle's medium (DMEM) containing 0.01% DNase I. To exclude macrophages and fibroblasts, respectively, filtration, centrifugation, and resuspension were processed for cell plating on mouse IgG-coated (0.75 mg/ml) Petri dishes and then plating on cell culture dishes, each at 37 °C for 60 min. The last cell isolates were cultivated in Ham's F12 culture medium after being seeded onto Type I collagen-coated dishes. After 48 h, most cells were attached. These cells were used for apoptosis assay, invasion assay, immunocytochemistry analysis, and mRNA analysis. The medium was replaced with serum-free X-VIVO 10 medium (04-380Q, Lonza, Walkersville, MD, USA) and maintained for 24 h to obtain supernatants for ELISA analysis. The purity of ATII cells isolated from lungs of naive mice using this method was consistently greater than 90%, as determined through immunofluorescence staining targeting prosurfactant protein C (pro-SP-C) (Yang et al. 2014; Jung et al. 2019; Lee et al. 2017). We previously published confocal microscopy images of purified ATII cells obtained with LSM5 PASCAL (Carl Zeiss, Jena, Germany) (Lee et al. 2017).

### Isolation of lung fibroblasts

Mice were used to isolate primary lung fibroblasts, which were then purified using modified techniques outlined by Akamatsu et al. (2013) and Lee et al. (2017). In summary, the mouse lungs were divided into tiny fragments, finely chopped, and then incubated for 90 min in DMEM supplemented with 5% fetal bovine serum (FBS) using an enzymatic digestion method that included DNase I. Following their passage through 100 and 40 μm pores (SPL Life Sciences, Pocheon-si, Korea), the cells underwent centrifugation, washing, and three days of growth in 6-cm dishes supplemented with 10% FBS-containing DMEM. For mRNA analysis and invasion experiments, confluent cells from the first passage were used. The fibroblast cultures consistently exhibited a purity surpassing 90%, as morphologically confirmed by their characteristic spindle shape and features. Validation was further ensured through the expression of the fibroblast marker vimentin (Fig. S1a) (Donaldson 2015).

### Preparation of alveolar macrophages

Alveolar macrophages were obtained using established protocols with minor modifications (Lee et al. 2017). Briefly, suspended alveolar macrophages were determined to be over 95% viable through trypan blue dye exclusion. Following 1 h incubation in 12-well plates (5 × 105 cells per well) with serum-free X-VIVO 10 medium, three consecutive washes were used to remove nonadherent cells. Around 90–95% of the cells adherent to the plastic surface displayed morphological features consistent with macrophages. The identification and purity of macrophages were further confirmed through immunostaining with Mac3 (Fig. S1b).

### Quantitative real-time PCR (qRT-PCR) analysis

Total RNA was isolated from lung cells and tissue using the Easy Spin RNA extraction kit (Intron, Seongnam Gyeonggi-do, South Korea) in compliance with the manufacturer's suggested methods. Then, the cDNA was synthesized using the ReverTraAce qPCR RT Master Mix (Toyobo, Osaka, Japan). Next, gene expression was assessed through qRT-PCR utilizing the StepOnePlus system (Applied Biosystems, Life Technologies, Carlsbad, CA, USA). Thermo Fisher Scientific's Primer Express program was used to create primer sets for PCR amplifications. Following a normalization process using hypoxanthine–guanine phosphoribosyl transferase (HPRT), fold differences between the obtained gene expression values and the control group were reported. The primer sequences employed for the amplification of the target genes can be found in Table S2.

### Western blot analysis

On 6–8% sodium dodecyl sulfate–polyacrylamide gels (#161–0158, Bio-Rad Laboratories, Hercules, CA, USA), samples of lung tissue homogenate and whole cell lysates were kept apart. Next, using a Bio-Rad Laboratories electrophoretically transfer apparatus, the separated proteins were placed onto nitrocellulose membranes. After that, blocking was done with 3% bovine serum albumin in tris-buffered saline at room temperature for 60 min. The membranes were probed for 20 h using the designated primary antibodies, and then they were incubated for 30 min with secondary antibodies (1:1000). An enhanced chemiluminescence detection kit (Thermo Scientific) was used for the detection process. Table S1 contains information on the antibodies that were used.

### Invasion assay

Following the manufacturer's instructions, cell invasion was measured using Transwell chambers (Corning Inc., Corning, NY, USA) pre-coated with Matrigel matrix (300 μg/ml for primary ATII cells or 200 μg/ml for primary fibroblasts). In summary, serum-free DMEM was used to plate pre-incubated primary ATII cells and lung fibroblasts (5 × 10^4^ cells/well) in replicate wells in the upper chambers. The lower wells were fed with 10% FBS and DMEM. The plates were then placed in an incubator at 37 °C for 48 h. Following a 4% paraformaldehyde treatment, the non-invading cells were scraped from the upper surface of the membrane using a cotton swap. The cells on the bottom surface were stained with 0.1% crystal violet, and distilled water was used to rinse the cells. For every sample (10 × magnification), three randomly chosen microscopic images from triplicate wells were recorded and measured.

### Immunocytochemistry

Following confluency, glass coverslip-cultured cells were permeabilized with 0.1% Triton X-100 (Sigma-Aldrich) after being fixed with 4% paraformaldehyde. During the course of an 18 h incubation period at 4 °C, each primary antibody collected target proteins. Donkey anti-rabbit IgG coupled with fluorescence (Jackson ImmunoResearch, West Grove, PA, USA) was used to identify and visualize the collected proteins. After staining, photographs of the cells were captured with a confocal microscope (LSM5 PASCAL, Carl Zeiss, Jena, Germany), utilizing the mounting medium Vectashield contains 4′,6-diamidino-2-phenylindole (DAPI, Vector Laboratories, Burlingame, CA, USA). Table S2 contains information on the sources of the antibodies and the dilution ratios.

### Immunohistochemistry

Tissues immersed in paraffin and fixed in formalin were sectioned at a thickness of 4 μm. Slides were rehydrated with graded ethanol solutions in distilled water after being deparaffinized twice with xylene. Deposition of collagen was assessed using Masson's trichrome staining. The extent of fibrosis in the entire lung was quantified employing the Ashcroft scoring method (Ashcroft et al. 1988). Scores varying between 0 (indicating normal lung) and 8 (representing complete fibrosis) were used to determine the degree of fibrosis. Sections were incubated at room temperature with primary antibodies against α-SMA, S100A4, cleaved caspase-3, surfactant protein–C (SPC), or control rabbit IgG in order to perform immunofluorescence analysis. A confocal microscope was used to image every slide.

### Apoptosis assay in isolated ATII cells

Apoptosis was assessed using a propidium iodide (PI)/annexin V-FITC staining kit (BD Biosciences, San Jose, CA, USA). 5 μl of FITC-conjugated annexin V and 5 μl of PI were incubated in the dark with primary ATII cells for 15 min at room temperature. Flow cytometry (ACEA NovoCyte, San Diego, CA, US) was then used to identify the cells expressing for FITC-conjugated annexin V. The NovoExpress software 1.5 was used to process the data. Additionally, primary ATII cells were stained in compliance with the guidelines provided by the manufacturer using a terminal deoxynucleotidyl transferase dUTP nick end labeling (TUNEL) kit (Roche, Basel, Switzerland). The process of quantifying TUNEL-positive cells comprised a blinded hand count of TUNEL-positive cells per high-power fields (HPFs) per slice. The resulting values were averaged for each mouse.

### Enzyme-linked immunosorbent assay (ELISA)

Analysis of PGE_2_ and PGD_2_ was conducted on BAL fluid and cell culture supernatants using EIA kits (Assay Designs, Ann Arbor, MI, US). Furthermore, active transforming growth factor-β1 (TGF-β1) (BioLegend, San Diego, California, US), hepatocyte growth factor (HGF), and Gas6 (R&D Systems) were evaluated using ELISA kits in accordance with the manufacturer's instructions.

### Measurement of hydroxyproline

Following the instructions provided by the manufacturer, the amount of hydroxyproline in the lung was measured using an Abcam hydroxyproline assay kit.

### Statistical analysis

The data is shown as mean ± S.E.M. Analysis of variance was used to perform multiple comparisons, and when necessary, Tukey's post hoc test was used. A two-tailed Student's t-test was used for comparisons between two sample means. A *P*-value of less than 0.05 was considered statistically significant. All data analyses were conducted using Graph Prism 5 software (GraphPad Software Inc., San Diego, CA, US). Every group in an in vivo study contained a minimum of three mice. Prior to randomization and the experimental intervention, no animal was excluded. Sample allocation to different groups was done using a random number method.

## Results

### rGas6 administration inhibits mesenchymal transition and invasion of ATII cells after BLM treatment

**Fig. 1:**
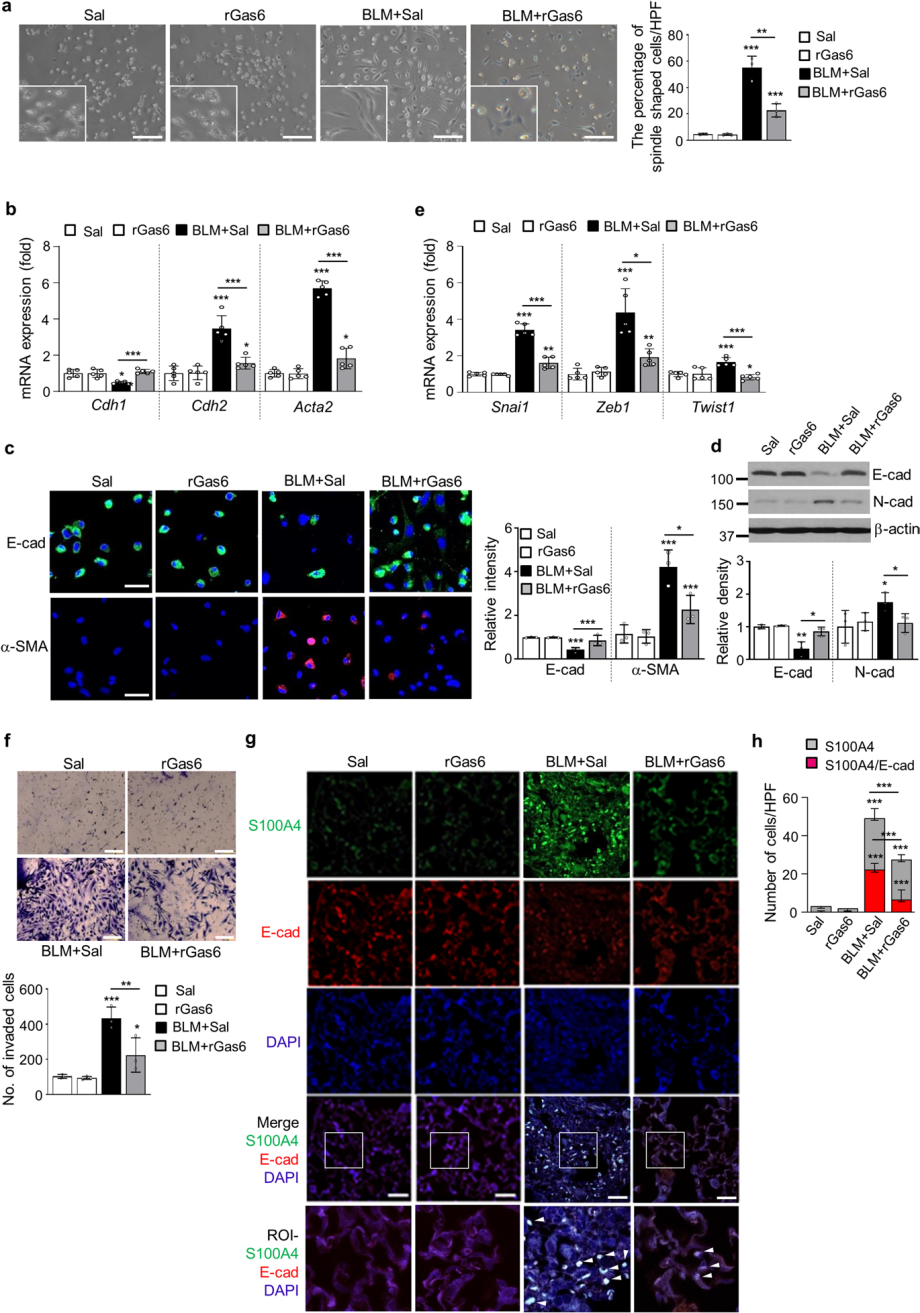
**Inhibition of EMT and primary ATII cell invasion by rGas6 administration.** Mice were intratracheally instilled with BLM (5 U/kg). Either rGas6 (50 μg/kg) or saline (Sal) was intraperitoneally administered 1 day before BLM treatment and once every 2 days thereafter. Mice were euthanized 14 days after BLM treatment. (**a**) Left: morphological changes in isolated ATII cells (Scale bars: 100 μm). Representative images are shown from three replicates per condition with cells pooled from two mice per replicate. Right: percentage of spindle shaped cells/high-power fields (HPF). (**b**) qRT-PCR of EMT markers in ATII cell samples. (**c**) Left: immunofluorescence staining for E-cadherin (green) and α-SMA (red). Right: quantification of proteins in ATII cells. Original magnification: 400 × . Scale bars: 20 μm. Imaging medium: Vectashield fluorescent mounting medium containing DAPI. (**d**) Immunoblot analysis of E-cadherin and N-cadherin in lung homogenates. Below: Densitometric analysis of each band normalized to that of β-actin. Values represent the means ± S.E.M. from three mice per group. (**e**) qRT-PCR of *Snail1, Zeb1, and Twist1* in ATII cell samples. (**f**) Phase-contrast microscopy and quantification of invaded ATII cells. Scale bars: 100 µm. *P < 0.05, **P < 0.01, ***P < 0.001 compared with control or for BLM + Sal vs. BLM + rGas6. Data were obtained from three (**c** *right*, **f** *below*) or five replicates (**b**, **e**) per condition with cells pooled from two mice per replicate. Data are shown as the means ± S.E.M. (**g**) Immunofluorescence staining for E-cadherin (red), α-SMA (red), or S100A4 (green) in lung sections. Arrowheads indicate colocalization of E-cadherin in lung fibroblasts. Imaging medium: Vectashield fluorescence mounting medium containing DAPI. Scale bars: 20 μm. Representative images were obtained from three mice in each group. (**h**) Graph representing the number of S100A4/E-cadherin double-positive cells compared with the total S100A4-positive cell population in lung parenchyma. Mean of five HPFs/section ± S.E.M. from three mice in each group

In our prior investigation (Lee et al. 2017), we developed an experimental approach for isolating primary ATII cells from mouse lung tissues, following the methodology outlined in a previous study (Bortnick et al. 2003). Through this approach, isolation of ATII cells was carried out from the lung tissues of mice subjected to BLM treatment. We found that approximately 53% of the isolated ATII cells 14 days post-BLM treatment exhibited a spindle-shaped morphology compared with those from control mice, when plated on a culture dish (Fig. 1a). The EMT process converts tightly adherent cells into an elongated, motile phenotype and has been linked to the progression of organ fibrosis (Kasai et al. 2005). Here, we demonstrate that rGas6 administration changed the morphology of isolated ATII cells a stretched, fibroblast-like morphology to a characteristic rounded shape at 14 days post-BLM treatment (Fig. 1a) and inhibited the mRNA expression profile of EMT markers (Fig. 1b). These actions induced a reduction of E-cadherin mRNA levels and enhancement of mRNA levels of N-cadherin and α-SMA, indicators of mesenchymal features, at 14 days post-BLM treatment. Immunofluorescence was conducted with monoclonal antibodies against E-cadherin (labeled in green) and α-SMA (labeled in red) to confirm alterations in EMT marker proteins. rGas6 treatment reversed decreased E-cadherin expression and increased α-SMA expression in primary ATII cells by BLM treatment (Fig. 1c). Similarly, 14 days post-BLM treatment, rGas6 prevented BLM-induced changes in the protein expression levels of N- and E-cadherin (Fig. 1d). The transition from epithelial to mesenchymal characteristics is orchestrated through the cooperative efforts of the Basic helix-loop-helix, ZEB, and Snai transcription factor families (Gonzalez and Medici 2014). Thus, 14 d following BLM treatment, we investigated if rGas6 injection reduces the expression of these transcription factors in primary ATII cells. ATII cells in the BLM + rGas6 group had considerably lower levels of *Snai1*, *Zeb1, and Twist1* mRNA than cells in the group that received BLM alone (Fig. 1e). The adoption of a mesenchymal phenotype by epithelial cells is linked to heightened invasive properties (Lamouille et al. 2014). Thus, Transwell invasion assays were conducted to evaluate cellular invasiveness in response to a chemoattractant gradient. Our data showed that rGas6 administration inhibited the invasive ability of primary ATII cells at 14 days after BLM treatment (Fig. 1f).

To validate the inhibitory impact of rGas6 on the EMT process, we conducted double immunofluorescence staining for E-cadherin and S100A4 in lung tissue. The noted existence of cells positive for both E-cadherin and S100A4 indicates an epithelial origin and a potential transitional phase of EMT. (Fig. 1g). Approximately 45% of fibroblasts positive for S100A4 were originated from lung epithelium at 14 days following BLM treatment (Fig. 1h), suggesting an obvious EMT occurrence after BLM treatment. Notably, both the quantity of fibroblasts originating from epithelial cells and the amount of S100A4 expression were reduced following Gas6 administration (~ 24% cells positive for both markers). When combined, these findings provide credence to the in vivo studies showing that rGas6 injection prevents lung fibrosis caused by BLM from progressing through the EMT stage.

### rGas6 administration inhibits apoptosis of ATII cell during lung fibrosis

**Fig. 2:**
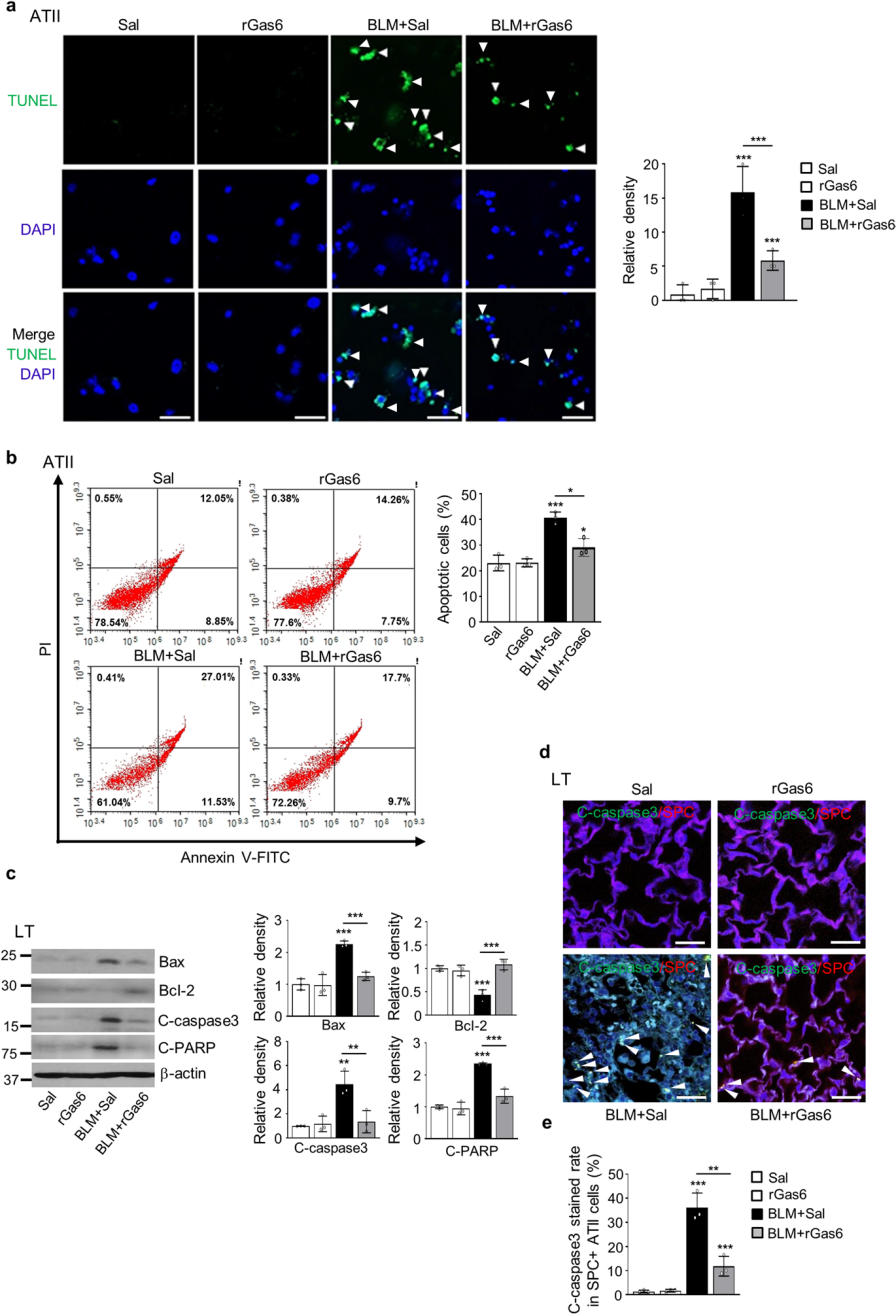
**Inhibition of apoptosis in ATII cells by rGas6 administration.** The experimental design was as described in Fig. 1. Mice were euthanized 14 days after BLM treatment. (**a**) Left: Representative TUNEL-stained and fixed ATII cells (original magnification: 400 ×). Positive staining depicted in green. Nuclei were observed by DAPI staining. Scale bars: 20 μm. Right: Quantitation of the number of TUNEL-positive cells (number/HPF) in the different groups. (**b**) The cell viability in primary ATII cells was measured by flow cytometry after annexin V-FITC/PI dual staining. Apoptotic cells were quantified as the sum of the percentages of cells in the early and late stages of apoptosis. **P < 0.01, ***P < 0.001 compared with control or for BLM + Sal vs. BLM + rGas6. Data were obtained from three replicates per condition with cells pooled from two mice per replicate (**a** *right*, **b** *right*). The data are shown as the means ± S.E.M. (**c**) Immunoblot analysis of Bax, Bcl-2, cleaved caspase-3, and cleaved PARP in lung homogenates. Below: Densitometric analysis of each band normalized to that of β-actin. (**d**) Representative confocal images of lung sections stained with an anti-SPC antibody (red), anti-cleaved caspase-3 antibody (green), and DAPI (blue) (*left*). Original magnification: × 400. Scale bars = 20 μm. Quantification of cleaved caspase-3 staining in SPC.^+^ ATII cells (*right*). The values represent the means ± S.E.M. of results from three mice from each group. **P < 0.01 compared with Sal control or for BLM + Sal vs. BLM + rGas6

In addition to EMT, many studies have shown that injury and apoptosis of alveolar epithelial cells are important early features of IPF (Todd et al. 2012). Using a TUNEL assay and flow cytometry analysis, we examined whether rGas6 administration attenuates apoptosis of primary ATII cells in the BLM-induced fibrotic phase. Similar to the findings from others studies (Safaeian et al. 2013), apoptosis of ATII cells were significantly enhanced at 14 days after BLM treatment (Fig. 2a and b). However, rGas6 decreased the count of TUNEL-positive ATII cells per HPF 14 days after BLM treatment (Fig. 2a). Additionally, rGas6 suppressed the apoptosis levels of primary ATII cells 14 days post-BLM treatment according to annexin V/PI staining and flow cytometry (from 28.3% to 17.7%) (Fig. 2b). In the lung tissue of the group subjected to both BLM and rGas6 treatment, the protein levels of apoptosis-related markers, including Bax, cleaved caspase-3, and cleaved PARP, were found to be lower than the group treated with BLM alone. However, the protein levels of antiapoptotic marker Bcl-2 were recovered in the BLM + rGas6 group (Fig. 2c). Furthermore, double immunofluorescence staining for cleaved-caspase-3 and SPC (a marker of ATII cells) in lung tissue was explored. When compared to the group treated with BLM alone, administration of rGas6 inhibited apoptosis of ATII cells (cleaved-caspase-3^+^/SPC^+^) 14 days after BLM treatment (Fig. 2d and e). Collectively, administration of rGas6 inhibited ATII cell apoptosis in the late fibrotic stage after BLM treatment.

### rGas6 administration inhibits fibroblast activation

**Fig. 3:**
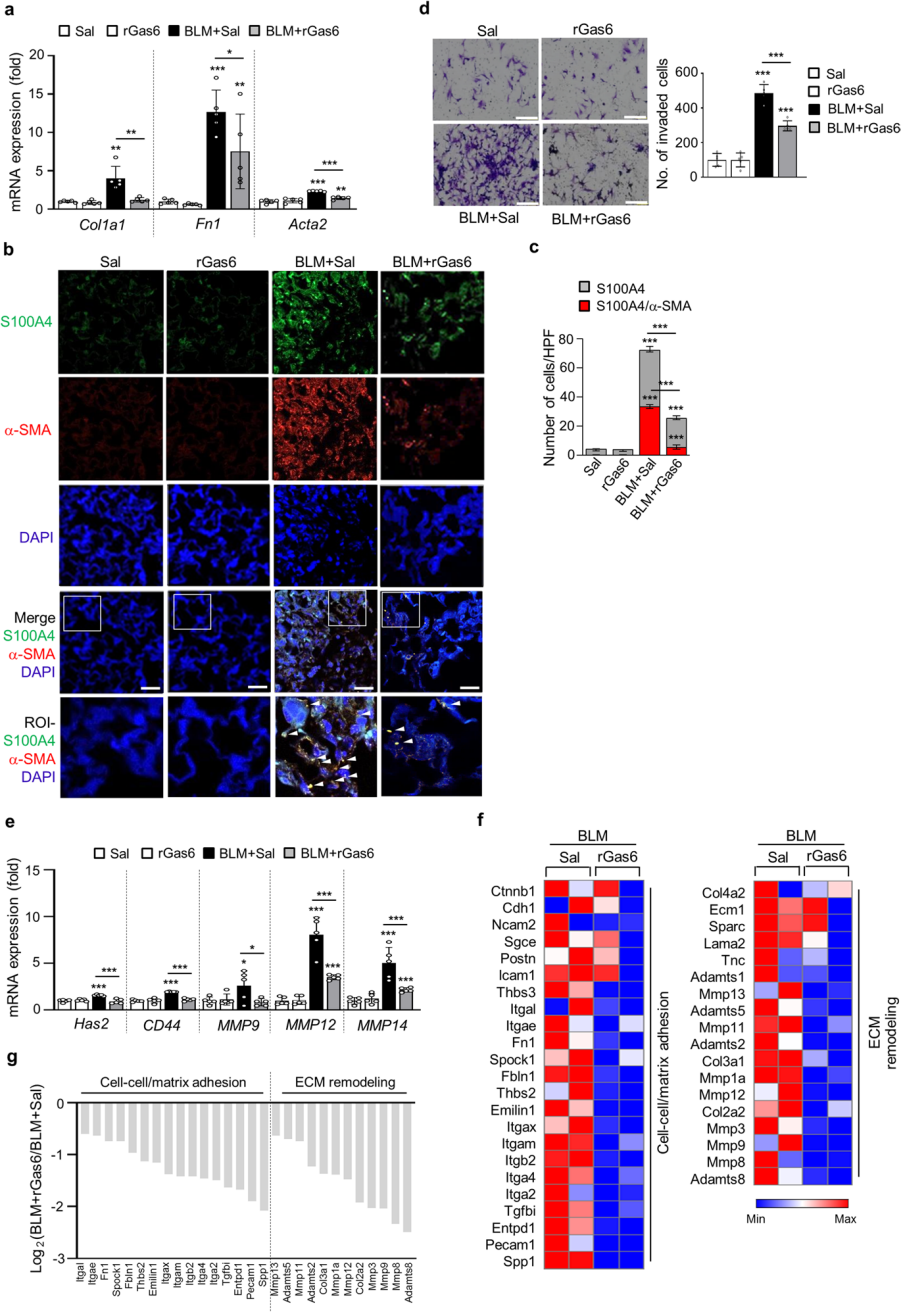
**Inhibition of fibroblast activation by rGas6 administration.** The experimental design was as described in Fig. 1. Mice were euthanized 14 (**a**, **d-g**) or 21 days (**b**) after BLM treatment. (**a**, **d–f**) Primary fibroblasts were isolated from murine lungs. (**a**) qRT-PCR of collagen type 1, fibronectin, and α-SMA in fibroblast samples. (**b**) Immunofluorescence staining for α-SMA (red) or S100A4 (green) was performed in lung sections. Arrowheads indicate colocalization of α-SMA in lung fibroblasts. Imaging medium: Vectashield fluorescence mounting medium containing DAPI. Scale bars: 20 μm. Representative images were obtained from three mice per group. (**c**) Graph representing the number of S100A4/α-SMA double-positive cells compared with the total S100A4-positive cell population in the lung parenchyma. Mean of five HPFs per section ± S.E.M. from three mice in each group. ***P < 0.001 compared with control or for BLM + Sal vs. BLM + rGas6. (**d**) Phase-contrast microscopy (*left*) and quantification of invaded fibroblasts (*right*) using Matrigel-coated Transwell plates. Scale bar: 100 µm. (**e**) qRT-PCR of *Has2*, *CD44*, *MMP9, MMP12,* and *MMP14* in fibroblast samples. *P < 0.05, **P < 0.01, ***P < 0.001 compared with control or for BLM + Sal vs. BLM + rGas6. Data were obtained from five replicates per condition with cells pooled from two mice per replicate (**a**, **d** *right*, **e**). The data are shown as the means ± S.E.M. (**f**) Selected heatmaps showing differentially expressed genes encoding adhesion and ECM molecules in primary lung fibroblasts between the BLM + Sal and BLM + rGas6 groups. Red: increased expression; blue: decreased expression. Data were obtained from two replicates per condition with cells pooled from two mice per replicate. (**g**) Relative expression levels of selected genes from PCR array profiling (**f**). Log_2_ fold-change values (ApoSQ-CAF CM vs. CAF CM, fold change > 1.5)

We determined whether rGas6 administration could inhibit lung fibroblast activation after BLM treatment. Enhanced expression of mRNA related to fibroblast activation markers, like collagen type 1, fibronectin, and α-SMA, after BLM treatment were reversed by rGas6 (Fig. 3a). We explored immunofluorescence to detect activated fibroblasts in lung tissue. Fibroblasts undergoing activation or differentiation into myofibroblasts were identified as cells positive for both S100A4 (a fibroblast marker) and α-SMA (a myofibroblast marker) (Piera-Velazquez and Jimenez 2019; Xia et al. 2017). Approximately 47% of myofibroblasts marked by the expression of α-SMA in the interstitium at 21 days post-BLM treatment (Fig. 3b and c). Lung sections showed a reduction in the quantity of double-stained cells (around 21%), and the protein expression of these markers upon administration of rGas6 at 21 days after BLM treatment. Significantly, the advancement of lung fibrosis relies on the conversion of fibroblasts into an aggressive myofibroblast phenotype. Hyaluronan synthase 2 (HAS2) and CD44 are upregulated in this particular phenotype, and matrix metalloproteinases (MMPs) and their inhibitors are coordinately expressed (Li et al. 2011). The present study showed that BLM-induced increases in invasion in isolated lung fibroblasts were substantially inhibited by rGas6 (Fig. 3d). In addition, enhanced mRNA levels of *Has2*, *CD44*, and *MMPs*, including *MMP9*, *MMP12*, and *MMP14*, in primary lung fibroblasts following BLM treatment were reversed by administration of rGas6 (Fig. 3e). These data suggest that rGas6 administration restrains the aggressive characteristics of stimulated fibroblasts by downregulation of these molecular markers.

To gain further understanding of the mechanisms through which rGas6 inhibits fibroblast invasion in vivo, we conducted a comprehensive analysis of 84 genes associated with cell adhesion and remodeling of ECM, employing a targeted qRT-PCR array. Comparing the expression of twelve genes linked to cell adhesion between the groups treated with BLM plus rGas6 and the group treated only with BLM, the expression of these genes was reduced by more than two times, including *Spp1, Pecam1, Entpd1, Tgfbi, Itga2, Itga4, Itgb2, Itgam, Itgax, Emilin1, Thbs2, Fbln1* (Fig. 3f). Nine ECM remodeling-related genes, including *Adamts8, Mmp8, Mmp9, Mmp3, Col2a2, Mmp12, Mmp1a, Col3a1, Adamts2*, were also downregulated (> twofold) in the group that received both BLM and rGas6 treatment as opposed to those who received BLM treatment alone. These patterns of gene expression resembled those of non-invasive fibroblasts (Lovgren et al. 2011), suggesting that rGas6 administration inhibits the transition to an invasive myofibroblast phenotype induced by BLM.

### rGas6 administration suppresses lung fibrosis

**Fig. 4:**
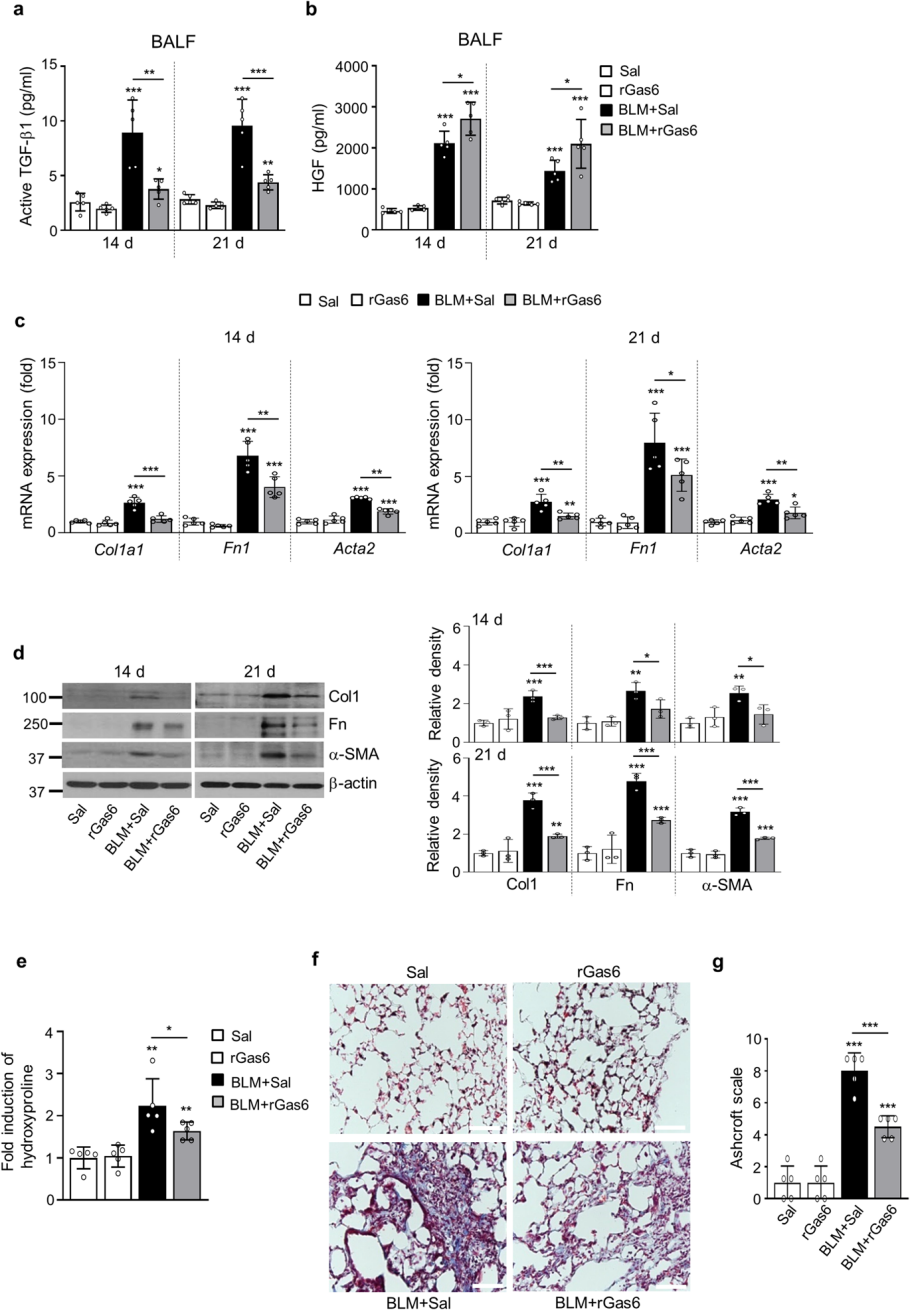
**Inhibition of lung fibrosis by rGas6 administration.** The experimental design was as described in Fig. 1. Mice were euthanized on days 14 and 21 after BLM treatment. (**a**, **b**) Levels of the active form of the TGF-β1 and HGF proteins in BAL fluid were quantified by ELISAs. (**c**) qRT-PCR of collagen type1, fibronectin, and α-SMA in lung tissue samples. (**d**) Left: Immunoblot analysis of the indicated proteins in lung homogenates. Right: Densitometric analysis of each band normalized to that of β-actin. (**e**) Collagen deposition in the whole lung was determined by measuring the hydroxyproline content on day 21. (**f**) Lung sections were visualized with Masson’s trichrome staining on day 21. Representative results from five mice per group are shown (scale bar: 50 μm). (**g**) Ashcroft scoring of the lung sections. The values represent the means ± S.E.M. of results from three (**d**) or five mice (**a**–**c**, **e**, **g**) in each group. *P < 0.05, **P < 0.01, ***P < 0.001 compared with Sal control or for BLM + Sal vs. BLM + rGas6

The concentrations or proportion of HGF/TGF-β1are likely pivotal in determining the equilibrium between damage and restoration in the later phases of fibrosis (Lee et al. 2012a, b). To confirm that the suppressive impacts of rGas6 on the EMT and fibroblast activation consequently prevent lung fibrosis, the levels of TGF-β and HGF, the major profibrotic and antifibrotic cytokines, respectively (Lee et al. 2012a, b), were examined following BLM treatment with or without rGas6. In BAL fluid 14 and 21 days after BLM treatment, rGas6 administration inhibited generation of the biologically active TGF-β, whereas HGF production was upregulated in comparison to the group that received BLM alone (Fig. 4a and b). At 14 and 21 days following BLM treatment, rGas6 substantially prevented the increase of mRNA and protein levels of myofibroblast phenotypic markers in lung tissue, such as collagen type 1, fibronectin, and α-SMA (Fig. 4c and d). Importantly, accumulation of collagen in lung tissue, assessed through hydroxyproline content, at 21 days after BLM treatment was substantially attenuated by rGas6 (Fig. 4e). Moreover, Masson’s trichrome staining illustrated a decrease in interstitial areas stained with collagen, accompanied by impaired alveolar structures 21 days after BLM treatment, indicating the protective effects of rGas6 (Fig. 4f). Histopathological assessment of pulmonary fibrosis was conducted employing the established Ashcroft scoring system (Ashcroft et al. 1988). The fibrotic score significantly decreased in the group receiving BLM and rGas6 in comparison to the group receiving BLM alone (Fig. 4g). Collectively, these findings imply that the administration of rGas6 inhibits EMT and fibroblast activation, thereby reducing ECM accumulation and preventing extensive lung injury in this BLM-induced murine lung fibrosis model.

### *rGas6 administration increases Gas6/Axl signaling events, including COX-2-derived PGE*_*2*_* and PGD*_*2*_* production*

Notably, Gas6 induction has been demonstrated in several fibrosis diseases, including IPF and liver fibrosis (Espindola et al. 2018; Bárcena et al. 2015). In particular, patients with IPF have higher levels of Gas6 expression in both mRNA and protein, compared to normal lung samples and fibroblasts, respectively (Espindola et al. 2018). This trend was consistently observed in our study, where protein levels of Gas6 increased in BAL fluid and in the conditioned media from primary ATII cells and alveolar macrophages of mice lungs post-BLM treatment (Fig. S2a). Moreover, in both primary ATII cells and lung tissue samples from mice, there was an increase in Gas6 expression at the mRNA and/or protein levels following BLM treatment (Fig. S2b–d). We further examined whether rGas6 administration enhances further Gas6 production in the lung post-BLM treatment. The Gas6 protein abundance was not further enhanced in BAL fluid and in the culture media from primary ATII cells and alveolar macrophages by administration of rGas6 (Fig. S2a). In addition, when compared to the group treated with BLM alone, the mRNA and/or protein levels of Gas6 in primary ATII cells and lung tissue in the group treated with both BLM and rGas6 did not increase further (Fig. S2b–d). Collectively, these findings indicate that rGas6 administration does not induce further increases in endogenous Gas6 production in ATII cells and lung tissue.

**Fig. 5:**
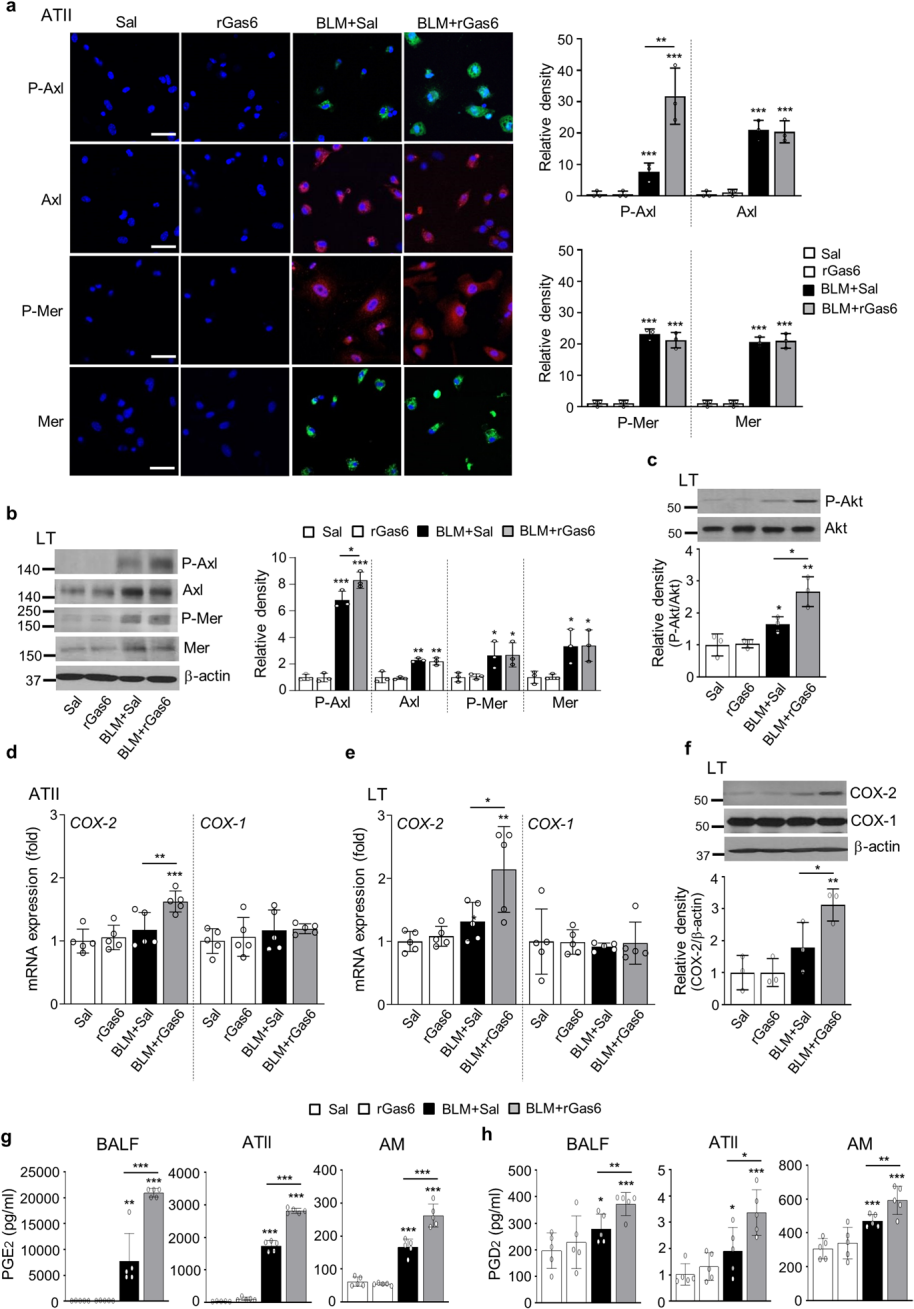
**Axl activation and COX-2-derived PGE**_**2**_** and PGD**_**2**_** production induced by rGas6 administration.** The experimental design was as described in Fig. 1. Mice were euthanized on day 14 after BLM treatment. (**a**) Left: Immunofluorescence staining for phospho-Axl (green), total Axl (red), phospho-Mer (red), and total Mer (green) in primary ATII cells. Images were captured at 400 × magnification. Right: Quantification of phospho-Axl, total Axl, phospho-Mer, and total Mer staining in ATII cells. Imaging medium: Vectashield fluorescence mounting medium containing DAPI. Scale bars: 20 μm. Data were obtained from three replicates per condition with cells pooled from two mice per replicate. (**b**) Left: Immunoblot analysis of total/phospho-Axl and total/phospho-Mer in lung tissue homogenates. Right: Densitometric analysis of each band normalized to that of β-actin. (**c**) Immunoblot analysis of total/phospho-Akt in lung tissue homogenates. Below: Densitometric analysis of each band normalized to that of total Akt. Data are from independent experiments with three mice per group (mean ± S.E.M.). (**d**, **e**) qRT-PCR of *COX-2* and *COX-1* in ATII cells and lung tissue samples. (**f**) Immunoblot analysis of COX-2 and COX-1 in lung tissue homogenates. Below: Densitometric analysis of each band normalized to that of β-actin. (**d**) Data were obtained from five replicates per condition with cells pooled from two mice per replicate. Data were obtained from independent experiments with five (**e**) or three (**f**) mice per group. (**g**, **h**) PGE_2_ or PGD_2_ levels in BAL fluid (BALF, n = 5 mice) and culture supernatants from ATII cells and alveolar macrophages (AM) were measured using an enzyme immunoassay. (**h**) Data were obtained from five replicates per condition with cells pooled from two mice per replicate. Values represent the means ± S.E.M. *P < 0.05, **P < 0.01, ***P < 0.001 compared with Sal control or for BLM + Sal vs. BLM + rGas6

Next, we examined whether in vivo administration of rGas6 induces such activation of Axl and Mer events in BLM-induced fibrosis. The findings from immunofluorescence demonstrated that the total protein expression levels of Axl and Mer in primary ATII cells (red and green, respectively) were notably increased in the group received both BLM and rGas6 as well as in the group received BLM alone (Fig. 5a). However, the phosphorylation level of Axl in ATII cells was further enhanced by rGas6 compared with that in the group treated solely with BLM, whereas the Mer phosphorylation level was similar to that in the BLM only treated group. Alveolar macrophages showed similar results in the group treated with both BLM and rGas6 showing a greater increase in Axl phosphorylation than the group treated with BLM alone, and between these groups, there was no variation in Mer's phosphorylation levels (Fig. S3). Western blot analysis showed that Axl phosphorylation was further enhanced in lung tissue by administration of rGas6 compared with that in the BLM only treated group, and the Mer phosphorylation levels were comparable between these experimental groups (Fig. 5b). In addition, activation of a downstream molecule in Gas6/Axl signaling, Akt, was also further enhanced in lung tissue by administration of rGas6 in comparison to the group treated with BLM alone (Fig. 5c).

Next, 14 days after BLM treatment, we measured the COX-1 and COX-2 expression as well as the synthesis of PGE_2_ and PGD_2_. In comparison to the BLM alone treated group, the administration of rGas6 further elevated the COX-2 mRNA levels in primary ATII cells as well as the COX-2 mRNA and protein levels in lung tissue (Fig. 5d–f). However, the amounts of COX-1's mRNA and protein remained constant. When rGas6 was administered, the synthesis of PGE_2_ and PGD_2_ in BAL fluid as well as the culture supernatants of isolated ATII cells and alveolar macrophages was further increased in comparison to the BLM alone treated group (Fig. 5g and h). Taken together, these findings indicate that rGas6 administration significantly enhances Axl phosphorylation and production of COX-2-derived bioactive molecules, such as PGE_2_ and PGD_2_, in ATII cells and alveolar macrophages.

Next, we aimed to confirm an in vivo role of Axl activation after rGas6 administration for PGE_2_ and PGD_2_ synthesis by COX-2 using the Axl inhibitor BGB324. The ATII cells' COX-2 mRNA level and the PGE2 and PGD2 levels in their culture media were both elevated by rGas6 day 14 post-BLM treatment. These effects were reversed by co-administration with BGB324, as shown in Fig. S4a-c. However, when NS-398 was co-administered with rGas6, rGas6-induced increases in PGE_2_ and PGD_2_ production were downregulated in the culture media from ATII cells (Fig. S4b and c). Collectively, these data suggested that rGas6-induced increases in Axl activation in ATII cells induce COX-2-dependent PGE_2_ and PGD_2_ production, which are well known for their inhibitory roles in EMT and fibroblast activation, thus preventing lung fibrosis [41, 42].

### Gas6/Axl signaling events are required for inhibition of EMT and fibroblast activation

**Fig. 6:**
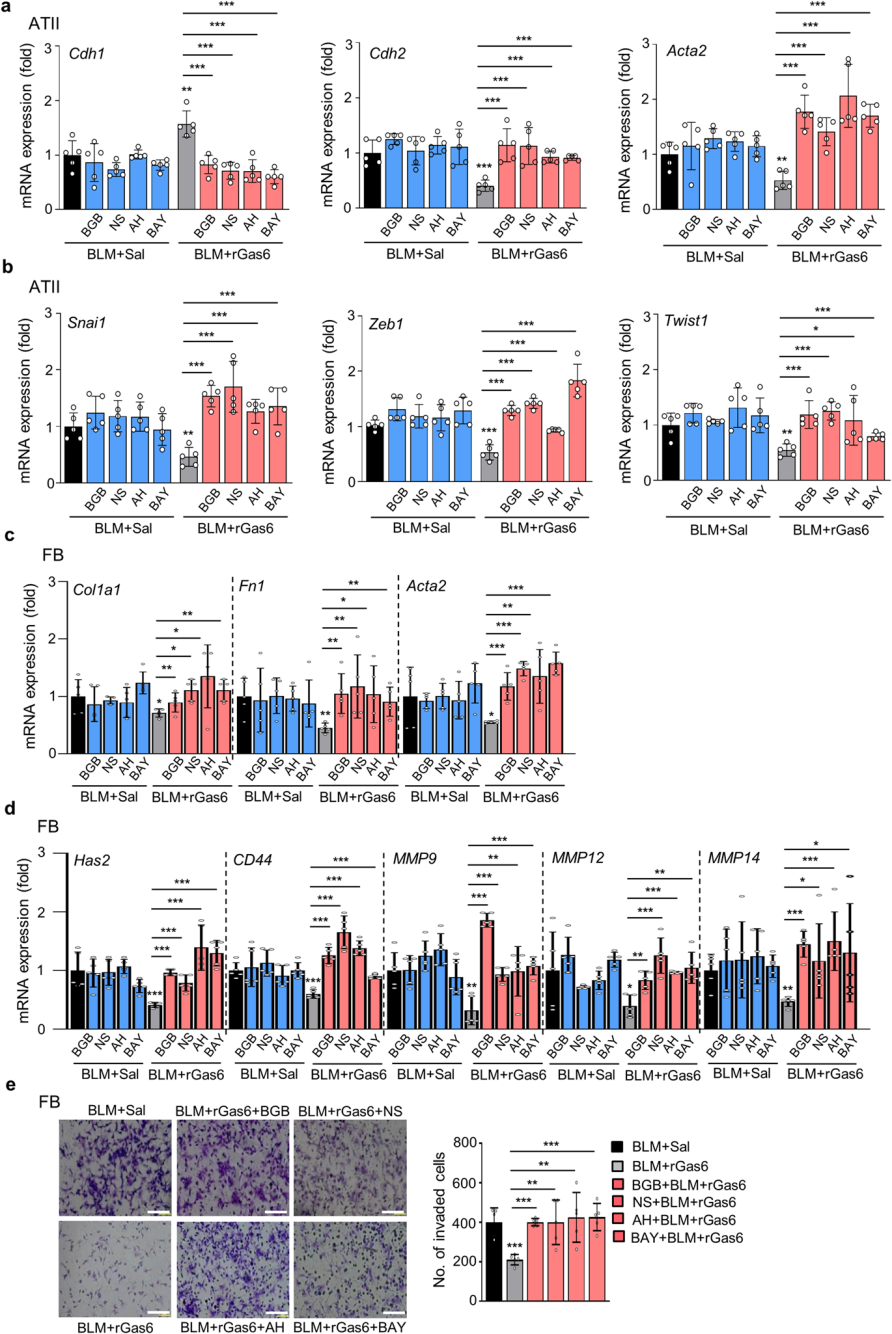
**Inhibition of EMT and fibroblast activation via Gas6/Axl signaling events.** Where indicated, the Axl inhibitor BGB324 (BGB, 5 mg/kg, *i.o.*), COX-2 inhibitor NS-398 (NS, 5 mg/kg, *i.o.*), EP1/EP2 inhibitor AH-6809 (AH, 5 mg/kg, *i.p.*), or DP2 inhibitor BAY-u3405 (BAY, 30 mg/kg, *i.p.*) was co-administered with rGas6 1 day before BLM treatment and then administered once/day (AH) or once every 2 days (BGB, NS, and BAY). Mice were euthanized 14 days following BLM treatment. (**a**, **b**) qRT-PCR of EMT markers and EMT-regulating transcription factors in primary ATII cells. (**c**, **d**) qRT-PCR of activated fibroblast markers and invasive myofibroblast-related molecules in primary lung fibroblasts. (**e**) Left: The cells were visualized by phase-contrast microscopy to analyze their invasive ability in Matrigel-coated Transwell assays. Scale bar: 100 µm. Right: The invaded fibroblasts were quantified by counting the number of cells adhering to the bottom surface of the upper chamber. *P < 0.05, **P < 0.01, ***P < 0.001 compared with BLM + Sal or for BLM + Gas6 vs. BLM + rGas6 + the inhibitor. Data were obtained from five replicates per condition with cells pooled from three mice per replicate (means ± S.E.M.)

To confirm the involvement of Gas6/Axl signaling pathways in mediating the inhibitory effects on BLM-induced EMT and fibroblast activation, the Axl selective inhibitor BGB324, the COX-2 inhibitor NS-398, and PGE_2_ and PGD_2_ receptor antagonists, including EP2/EP4 (AH-6809) and DP2 (BAY-u3405) antagonists, were co-administered with rGas6 1 day before BLM treatment and then administered once/day (AH-6809) or once every 2 days (BGB324, NS398, and BAY-u3405) for 2 weeks after BLM treatment. When BGB324, NS-398, AH-6809, or BAY-u3405 was administered together, the anti-EMT effects of rGas6 were significantly reduced. This included the inhibition of E-cadherin loss, the reduction of N-cadherin and α-SMA synthesis at the mRNA levels (Fig. 6a), and the restoration of *Snai1*, *Zeb1*, and *Twist1* mRNA levels in primary ATII cells 14 days after BLM treatment (Fig. 6b). However, these inhibitors alone had no effect in mice treated with BLM alone.

We also examined whether Gas6/Axl signaling events inhibit fibroblast activation using these inhibitors. Importantly, co-administration of inhibitors (BGB324, NS398, AH-6809, or BAY-u3405) inhibited the rGas6-induced reduction in the mRNA expression levels of activated fibroblast markers, such as collagen type 1, fibronectin, and α-SMA, as well as invasive fibroblast phenotype-mediating factors, such as *Has2*, *CD44*, *MMP9*, *MMP12*, and *MMP14*, in primary fibroblasts at 14 days post-BLM treatment (Fig. 6c and d). However, these inhibitors had no significant effect in mice treated with BLM alone. Indeed, the inhibition of fibroblast invasion by rGas6 administration was also counteracted by co-administration of these inhibitors. (Fig. 6e). Collectively, these findings suggest that Gas6/Axl signaling events consequently mediate rGas6-induced inhibition of the EMT process and fibroblast activation in BLM-induced lung fibrosis.

### *The progression of the EMT and the activation of fibroblasts are aggravated in Gas6*^*−/−*^* mice*

**Fig. 7:**
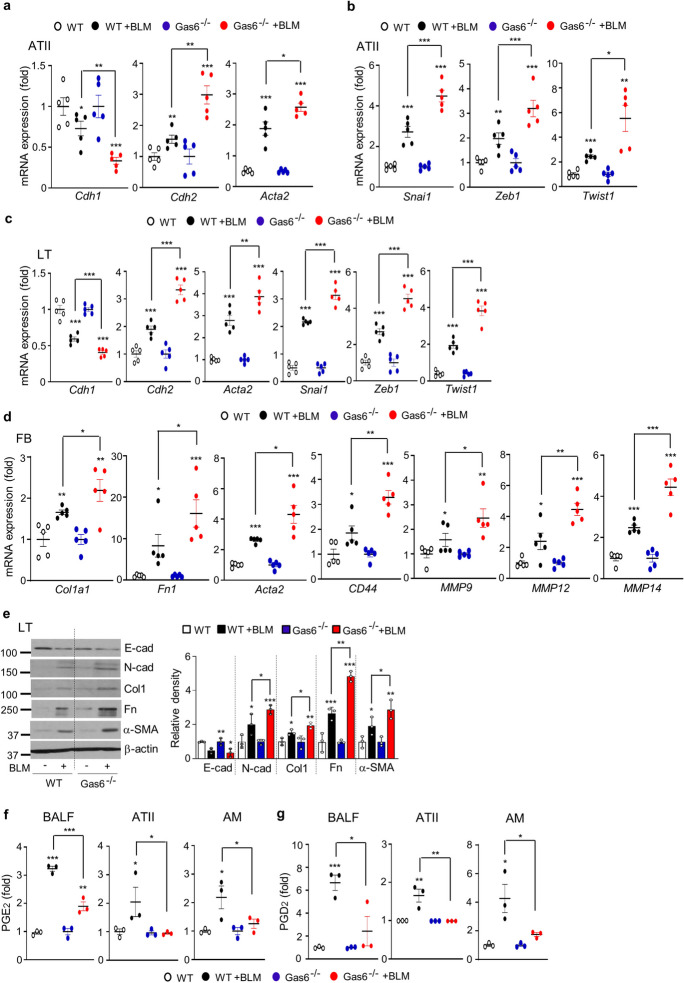
**Effect of Gas6 deficiency on EMT and fibroblast activation.** WT and GAS6^−/−^ mice were intratracheally instilled with BLM (5 U/kg). Mice were euthanized 14 days after BLM treatment. (**a**–**c**) qRT-PCR of EMT markers and EMT-regulating transcription factors in primary ATII cells (**a**, **b**) and lung tissue (**c**). (**d**) qRT-PCR of activated fibroblast markers and invasive myofibroblast phenotype-regulating molecules in primary fibroblasts. (**e**) Left: Immunoblot analysis of the indicated proteins in lung tissue. Right: Densitometric analysis of each band normalized to that of β-actin. (**f**, **g**) PGE_2_ and PGD_2_ levels in BAL fluid (BALF) and conditioned media of ATII cells and alveolar macrophages (AM) were measured using an enzyme immunoassay. *P < 0.05, **P < 0.01, ***P < 0.001 compared with Sal control or for WT + BLM vs. GAS6^−/−^ + BLM. Data were obtained from three (**f** and **g** *middle, right*) or five replicates (**a**, **b**, **d**) per condition with cells pooled from two mice per replicate (means ± S.E.M.). Values represent the means ± S.E.M. of results from three (**e** *right*, **f** and **g** *left*) or five mice (**c**) per group

**Fig. 8:**
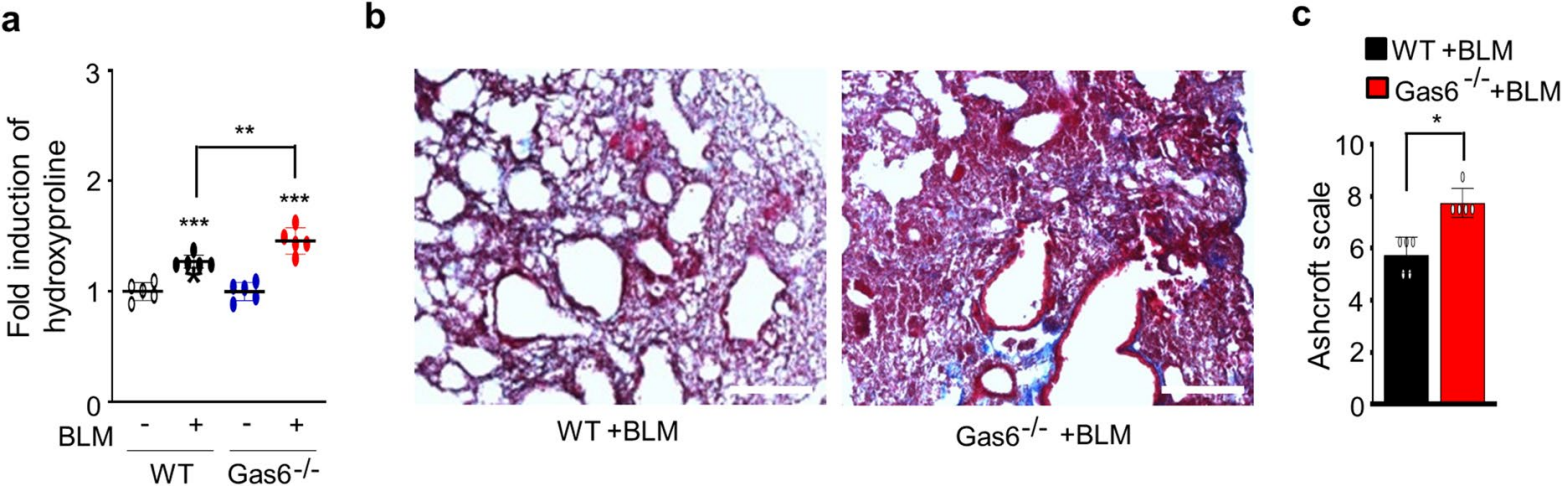
**Effect of Gas6 deficiency on collagen deposition in lung fibrosis.** WT and GAS6^−/−^ mice were intratracheally instilled with BLM (5 U/kg). Mice were euthanized 14 days after BLM treatment. (**a**) Collagen deposition in the whole lung was determined by measuring hydroxyproline content. (**b**) Lung sections were visualized with Masson’s trichrome staining on day 14. Representative results from three mice per group are shown (scale bar: 50 μm). (**c**) Ashcroft scoring of the lung sections. *P < 0.05, **P < 0.01, ***P < 0.001 compared with Sal control or for WT + BLM vs. GAS6^−/−^ + BLM. Values represent the means ± S.E.M. of results from three (**c**) or five mice (**a**) per group

To confirm the suppressive function of endogenous Gas6 in BLM-induced fibrosis, we examined changes in the EMT process, fibroblast activation, and hydroxyproline content using Gas6^−/−^ and wild-type (WT) control mice after BLM treatment. Using fluorescent immunocytochemical staining, we confirmed the loss of Gas6 in primary ATII cells of Gas6^−/−^ mice (Fig. S5a). Additionally, in both isolated ATII cells and lung tissue from Gas6^−/−^ animals with or without BLM treatment, endogenous Gas6 expression at the mRNA and/or protein levels was not detected (Fig. S5b-d). After 14 days of BLM treatment, ATII cells from Gas6^−/−^ mice exhibited modifications in EMT markers' mRNA levels caused by BLM, including α-SMA, N-cadherin, and E-cadherin, as well as increases in transcription factors that regulate EMT, including *Snai1*, *Zeb1*, and *Twist1*, when compared to WT control mice (Fig. 7a and b). Comparing lung tissue from Gas6^−/−^ mice to WT control mice, similar results were found regarding the mRNA expression levels of EMT markers and EMT-regulating transcription factors (Fig. 7c). Significant elevation in the mRNA levels of activated fibroblast markers, such as collagen type 1, fibronectin, and α-SMA, and invasive fibroblast phenotype-regulating molecules, such as *CD44*, *MMP9*, *MMP12*, and *MMP14*, were also observed in primary fibroblasts from Gas6^−/−^ mice at 14 days post-BLM treatment than in those from WT control mice (Fig. 7d). Additionally, BLM-induced alterations in markers related to EMT and fibroblast activation, including E-cadherin, N-cadherin, collagen type-1, fibronectin, and α-SMA, at the protein level were amplified in lung tissue from Gas6^−/−^ mice compared with that from WT mice (Fig. 7e). Simultaneously, BLM-induced the levels of PGE_2_ and PGD_2_ in BAL fluid and conditioned media from ATII cells and alveolar macrophages were reduced in Gas6^−/−^ mice (Fig. 7f and g), but the hydroxyproline amount in lung tissue from Gas6^−/−^ mice was further enhanced compared with that in WT control mice (Fig. 8a). Furthermore, interstitial regions marked by collagen deposition in interstitial areas with impaired alveolar structures, and fibrotic scores were additionally heightened in Gas6^−/−^ mice 14 days post-BLM treatment compared with those in WT control mice (Fig. 8b and c). Collectively, these data suggest Gas6-deficient mice exhibit exaggerated BLM-induced EMT and fibroblast activation, leading to further accumulation of collagen and intensified fibrosis, indicating a protective role of Gas6 against the progression of pulmonary fibrosis.

### In vitro* exposure of LA-4 cells to rGas6 inhibits myofibroblast phenotypic markers*

To ascertain the direct relationship between GAS6 signaling in ATII cells and the activation of lung fibroblasts, the conditioned medium from ATII-like lung (LA-4) epithelial cells treated with rGas6 (400 ng/ml) was applied to lung fibroblasts (MLg cells) with or without TGF-β1. Exposure to this conditioned medium inhibited TGF-β1-induced mRNA and protein expression of collagen type 1, fibronectin, and α-SMA (Fig. S6a-d). However, conditioned medium derived from LA-4 cells without exposure to rGas6 did not exhibit the inhibitory effect. These data indicate that the downregulation of fibroblast activation is contingent upon paracrines secreted from LA-4 cells stimulated with rGas6.

## Discussion

We hypothesized that in vivo administration of rGas6 could prevent EMT in ATII cells and fibroblast activation to consequently induce antifibrotic effects in BLM-induced lung fibrosis. We first demonstrated that rGas6 suppressed EMT, leading to changes in morphology and modulation of EMT markers. This included a reduction of E-cadherin and increases in N-cadherin, α-SMA, and key regulators of EMT, such as Snai1 and Zeb1, and Twist1*,* and invasion ability in primary ATII cells 14 days after BLM treatment. Notably, in human IPF tissue, there was colocalization observed between epithelial and mesenchymal markers (Lomas et al. 2012; Willis et al. 2005). Similarly, immunohistochemical fluorescence staining demonstrated a decrease in the count of cells exhibiting co-expression of E-cadherin/S100A4 and α-SMA/S100A4 after the administration of rGas6. This indicates an epithelial origin and a plausible transitional phase in the EMT. It is also suggested that alveolar epithelial cells may undergo a phenotypic transition via the EMT to become myofibroblasts and participate in the development of lung fibrosis. Repetitive lung injuries lead to aberrant activation of EMT pathways due to the inability of the alveolar epithelium to regenerate (Salton et al. 2019). Here, we demonstrate that rGas6 administration significantly suppressed primary ATII cell apoptosis in the late phase of BLM treatment. These changes were associated with reduction in the protein expression of proapoptotic markers, including Bax, cleaved caspase-3 and cleaved PARP and increase in an antiapoptotic marker, such as Bcl-2, in lung tissue following rGas6 administration.

Epithelial cells undergoing EMT subsequently communicates with the interstitial milieu, promoting the differentiation of myofibroblasts and the development of fibrosis (Harada et al. 2010). In addition to an anti-EMT effect, we also found that rGas6 inhibited fibroblast activation in BLM-induced fibrosis. Indeed, the enhanced mRNA expression levels of myofibroblast phenotype markers in primary fibroblasts were reduced by rGas6 administration. Moreover, immunohistochemical analysis of lung sections demonstrated that the count of cells exhibiting co-expression of α-SMA/S100A4 was reduced by rGas6. The Transwell assay results revealed that primary fibroblasts from the group treated with both BLM and rGas6 exhibited lower invasiveness compared to those from the group treated solely with BLM. Consistent with these findings of reverting myofibroblasts to an inactivated phenotype, rGas6 administration reduced the mRNA expression levels of *Has2*, *CD44*, *MMP9*, *MMP12*, and *MMP14,* which have been shown to promote cell invasion, in primary fibroblasts (Marrero-Diaz et al. 2009). Concomitantly, lung content of hydroxyproline, and collagen-stained interstitial area with damaged alveolar structures in lung sections during BLM-induced fibrosis were significantly reduced by rGas6 administration. These data support the antifibrotic effects of rGas6, demonstrating that concomitant inhibition of EMT and fibroblast activation by rGas6 may account for its effect in preventing the development of BLM-induced lung fibrosis. Notably, our in vitro findings highlight the direct relationship between Gas6 signaling in ATII cells and the activation of lung fibroblasts. Treatment of MLg fibroblasts with the rGas6-exposed LA-4 CM resulted in a reduction of the TGF-β1-induced upregulation of mRNA and protein expression associated with myofibroblast phenotypic markers, exemplified by type 1 collagen α2, fibronectin, and α-SMA.

It has been shown that the pathways involving COX-2/PGE_2_ and PGD_2_ block EMT in kidney and lung cells (Yoon et al. 2016; Zhang et al. 2006). Additionally, numerous investigations have indicated that PGE2 displays antifibrotic effects on various tissues by suppressing cell proliferation (Lama et al. 2002; Huang et al. 2007), migration (Kohyama et al. 2001), collagen expression and deposition (Huang et al. 2007), and fibroblast differentiation (Bärnthaler et al. 2020). These effects are facilitated through the cAMP/PKA signaling cascade when PGE_2_ binds to EP2 or EP4 receptors on fibroblasts. In addition, mice deficient in hematopoietic PGD synthase exhibited elevated collagen deposition in the lungs 14 days post-BLM treatment. (Kida et al. 2016). Findings from our earlier investigation illustrated that Gas6/TAM signaling triggers PI3K/Akt-STAT1 pathway for resolving inflammatory response (Kim et al. 2016). The JAK/STAT1 pathway has been demonstrated to upregulate COX-2 expression in lung epithelial cells (Lee et al. 2006). These data suggest that Gas6/TAM signaling at least partially involving PI3K/Akt-STAT1 pathway may be responsible for COX-2 induction in macrophages and epithelial cells. Here, we demonstrated that Axl phosphorylation, rather than Mer, was further enhanced in ATII cells and alveolar macrophages following rGas6 administration 14 days post-BLM treatment. Moreover, the Akt phosphorylation, a widely acknowledged downstream mediator of TAM signaling, in lung tissue was also further enhanced following rGas6 administration. Indeed, co-administration of BGB324, a small-molecule inhibitor of Axl, with rGas6 downregulated COX-2 expression as well as PGE_2_ and PGD_2_ production. This in turn mitigated the rGas6-induced suppression of the EMT process and fibroblast activation. Similarly, NS-398 as well as AH-6809 and BAY-u3405 could reverse the inhibition of EMT and fibroblast activation induced by rGas6. However, these findings are limited to demonstrating the effects of these inhibitors at the mRNA level in primary ATII cells and fibroblasts under the same experimental conditions. Taken together, our findings suggest that Gas6/Axl/Akt pathway possibly leads to the increased COX-2 transcriptional induction as well as PGE_2_ and PGD_2_ secretion to protect against the development of the EMT phenotype in ATII cells and the myofibroblast phenotype during BLM-induced lung fibrosis.

Data from our earlier in vitro investigation showed that PGE_2_ and PGD_2_ synthesis are induced by the Gas6-Axl pathway, which effectively prevents the abnormal development of EMT in LA-4 cells. This is accomplished by inhibiting TGF-β1-induced Akt and extracellular signal-regulated kinase (ERK)1/2 activation (Jung et al. 2019). Thus, in accordance with the results obtained from our pharmacological inhibition studies, it could be assumed that the antagonists blocking the activities of Axl, COX-2, and EP2 and DP2 receptors finally restore non-Smad TGF-β signaling pathways to suppress EMT process under the fibrotic conditions.

As expected, we found the upregulated Gas6 and Axl expression in lung cells and tissue, indicating that tissue homeostatic regulation during tissue repair and fibrosis may be potentiated upon BLM insult. Along with our findings, other studies also showed Gas6 and Axl expression were potentiated when triggered by noxious or inflammatory stimuli (Ekman et al. 2011; Gruber et al. 2014; Lemke and Rothlin 2008). Importantly, the defensive function of endogenous Gas6 in BLM-induced lung fibrosis was confirmed through investigations performed using mice lacking Gas6. In our investigation, we showed that the lack of Gas6 exacerbated lung fibrosis by causing an increased EMT process and fibroblast activation 14 day post-BLM treatment compared with that in WT control mice. Thus, our hypothesis was confirmed because Gas6 signaling is essential for preventing or blocking the progression of pulmonary fibrosis through inhibition of the EMT process and alveolar epithelial cell apoptosis as well as concomitantly restraining fibroblast activation. However, the antifibrotic action of Gas6 appears to conflict with that described in other reports. Particularly, targeting TAM receptors with BGB324 significantly attenuated the activation of fibroblasts from IPF lungs (Espindola et al. 2018). This discrepancy with our present study using Gas6^−/−^ mice might be explained by different fibrosis stages due to the use of different doses of BLM (1.7 U/kg vs. 5 U/kg) and limited numbers of experimental mice. However, the administration of exogenous protein S markedly reduced the levels of inflammatory and profibrotic markers to attenuate BLM-induced lung fibrosis (Urawa et al. 2016). Thus, further investigations using diverse in vivo lung fibrosis models across different species and disease states are necessary to fully understand the conflicting results concerning the anti- or pro-fibrotic hypothesis for Gas6.

## Conclusions

**Fig. 9:**
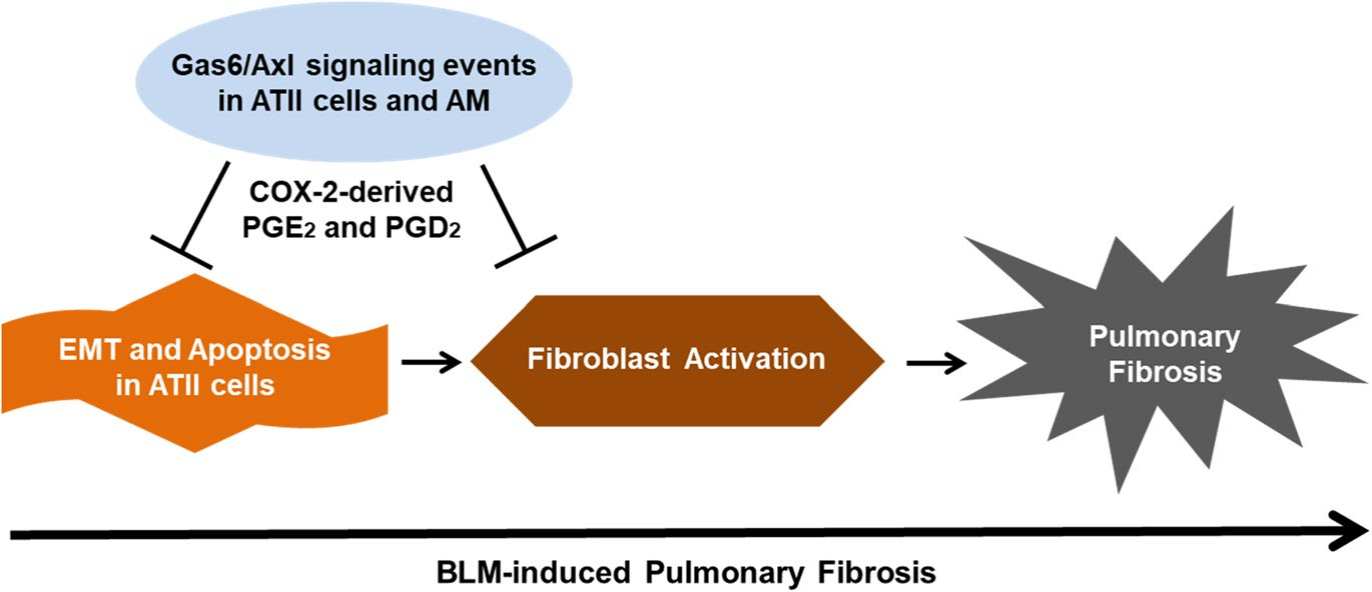
**A schematic diagram summarizing the role of Gas6/Axl signaling events for the prevention of lung fibrosis.** rGas6 inhibits EMT and apoptosis in ATII cells and concomitantly suppresses fibroblast activation, consequently preventing the development of BLM-induced lung fibrosis. This occurs through the activation of Axl signaling pathway, including COX-2-derived PGE_2_ and PGD_2_ production in ATII cells and alveolar macrophages

Here, we propose that rGas6 inhibits EMT and apoptosis in ATII cells and concomitantly suppresses fibroblast activation. This, in turn, hinders the advancement of lung fibrosis following BLM treatment. The Gas6/Axl signaling pathway may target ATII cells and alveolar macrophages, inducing production of PGE_2_ and PGD_2_ derived from COX-2. These prostanoids work in an autocrine and paracrine way to facilitate Gas6's anti-EMT and antifibrotic actions (Fig. 9). Furthermore, Gas6-deficient mice exhibited an intensified EMT in ATII cells and more activated myofibroblast-like phenotypes, consequently aggravating pulmonary fibrosis after BLM treatment.

## Supplementary Information

Below is the link to the electronic supplementary material.

Supplementary file1 (DOCX 925 KB)

## Data Availability

Data supporting the present study are available from the corresponding author upon reasonable request.
