# Supplementary material for: Administration of Gas6 attenuates lung fibrosis via inhibition of the epithelial-mesenchymal transition and fibroblast activation
Source: Cell Biol Toxicol. 2024 Apr 5;40(1):20. doi: 10.1007/s10565-024-09858-5 (PMC10997547; doi:10.1007/s10565-024-09858-5)

**Supplementary material**

**Administration of Gas6 attenuates lung fibrosis via inhibition of the epithelial-mesenchymal transition and fibroblast activation**

Ye-Ji Lee • Minsuk Kim • Hee-Sun Kim • Jihee Lee Kang

*Correspondence should be addressed to:

[jihee@ewha.ac.kr](mailto:jihee@ewha.ac.kr)

**
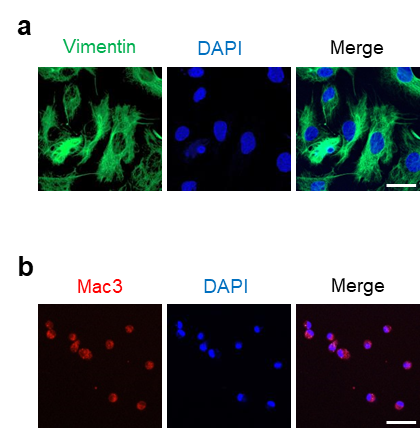
**

**Supplementary Figure 1**. Immunofluorescence staining for vimentin in primary isolated lung fibroblasts and for and Mac3 in alveolar macrophages from mice, respectively. Immunofluorescence staining for vimentin (green) in the isolated fibroblasts (**a**) and Mac3 (red) in alveolar macrophages (**b**) from naïve mice. Original magnification: 400×. Scale bars: 20 μm. Imaging medium: Vectashield fluorescent mounting medium containing DAPI. Results are representative of three independent experiments

**
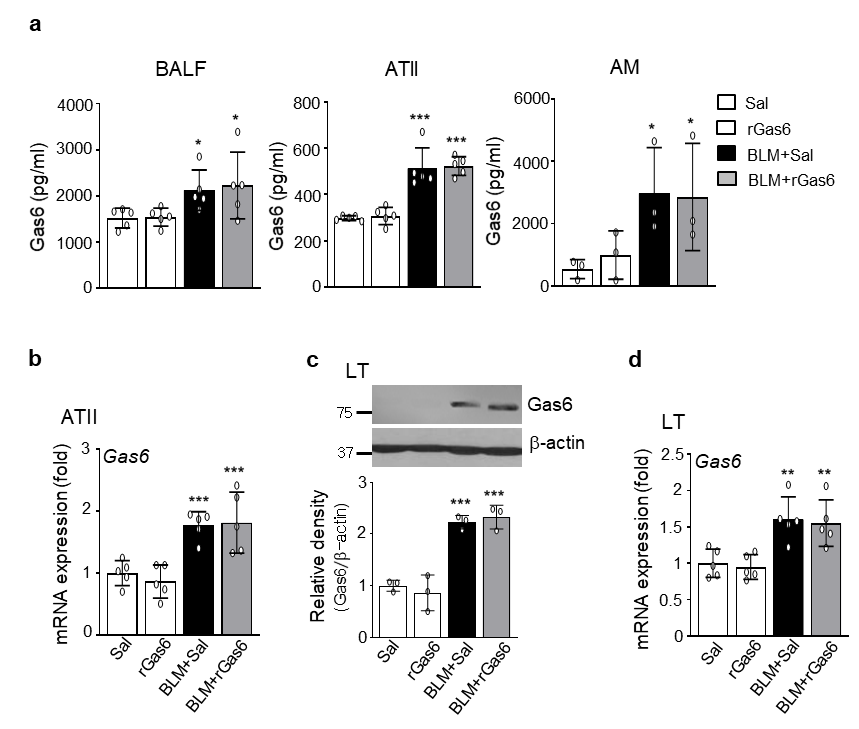
**

**Supplementary Fig. 2. Enhanced Gas6 expression in BLM-induced lung fibrosis.** Mice were intratracheally instilled with BLM (5 U/kg). Either rGas6 (50 μg/kg) or saline (Sal) was intraperitoneally administered 1 day before BLM treatment and once every 2 days thereafter. Mice were euthanized 14 days after BLM treatment. (**a**) ELISA of Gas6 in BAL fluid (BALF), culture supernatants from ATII cells, and alveolar macrophages (AM). (**b**) qRT-PCR of Gas6 in ATII cell samples. (**c**) Immunoblot analysis of Gas6 in lung tissue. Below: Densitometric analysis of each band normalized to that of β-actin. (**d**) qRT-PCR of Gas6 in lung tissue. **P* < 0.05, ***P* < 0.01, ****P* < 0.001 compared with Saline or rGas6. Data were obtained from three (**a** *right*) or five replicates (**a** *middle*, **b**) per condition with cells pooled from two mice per replicate (means ± SEM). Values represent the means ± SEM of results from three (**c** *below*) or five mice (**a**, **d**) per group.

**
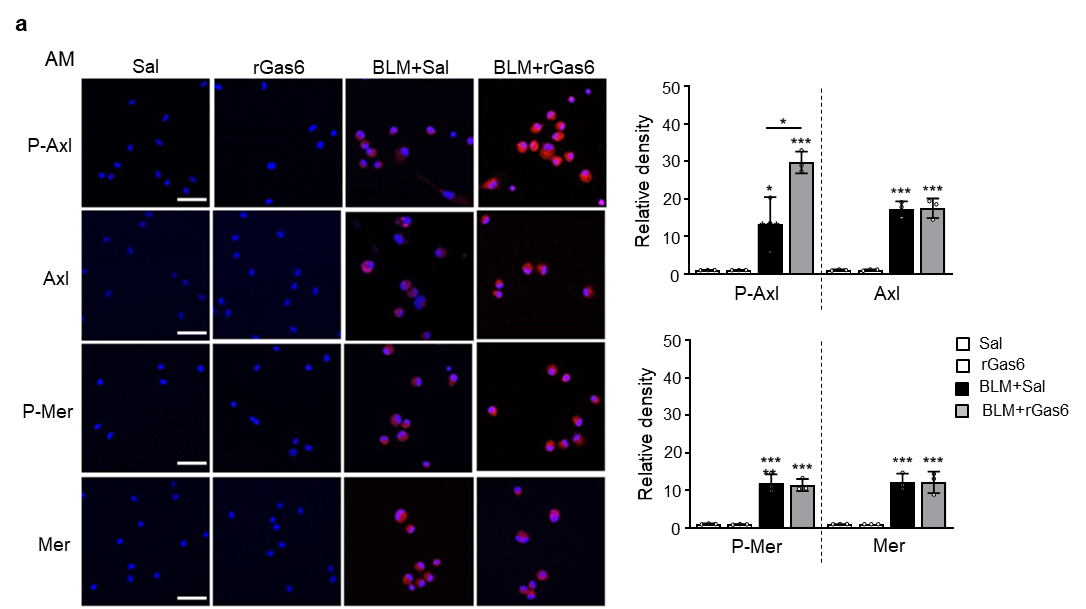
**

**Supplementary Fig. 3. Enhanced activation of Axl in alveolar macrophages induced by rGas6 administration.** Mice were intratracheally instilled with BLM (5 U/kg). Either rGas6 (50 μg/kg) or saline (Sal) was intraperitoneally administered 1 day before BLM treatment and once every 2 days thereafter. Mice were euthanized 14 days after BLM treatment. Left: Immunofluorescence staining for phospho-Axl (red), total Axl (red), phospho-Mer (red), and total Mer (red) in alveolar macrophages. Images were captured at 400× magnification. Right: Quantification of phospho-Axl, total Axl, phospho-Mer, and total Mer staining. Imaging medium: Vectashield fluorescence mounting medium containing DAPI. Scale bars: 20 μm. Data were obtained from three replicates per condition with cells pooled from two mice per replicate. Values represent the means ± SEM. **P* < 0.05, ****P* < 0.001 compared with saline control or rGas6.

**
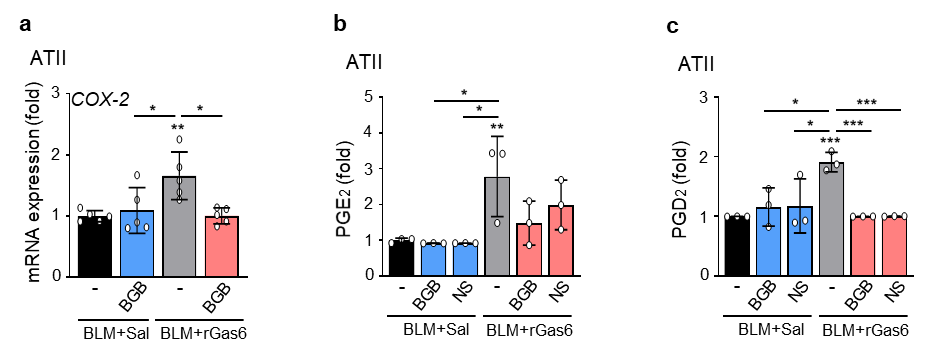
**

**Supplementary Fig. 4. Enhanced COX-2-derived PGE_2_ and PGD_2_ production induced by Gas6-Axl signaling.** Where indicated, the Axl inhibitor BGB324 (BGB, 5 mg/kg, *i.o.*) or COX-2 inhibitor NS-398 (NS, 5 mg/kg, *i.o.*) was co-administered with rGas6 1 day before BLM treatment and then administered once every 2 days thereafter. Mice were euthanized 14 days following BLM treatment. (**a**) qRT-PCR of *COX-2* in ATII cells. (**b, c**) PGE_2_ or PGD_2_ levels in culture supernatants from ATII cells were measured using an enzyme immunoassay. Data were obtained from three (**b**, **c**) or five replicates (**a**) per condition with cells pooled from two mice per replicate. Values represent the means ± SEM. **P* < 0.05, ***P* < 0.01, ****P* < 0.001 compared with saline control or rGas6.

**
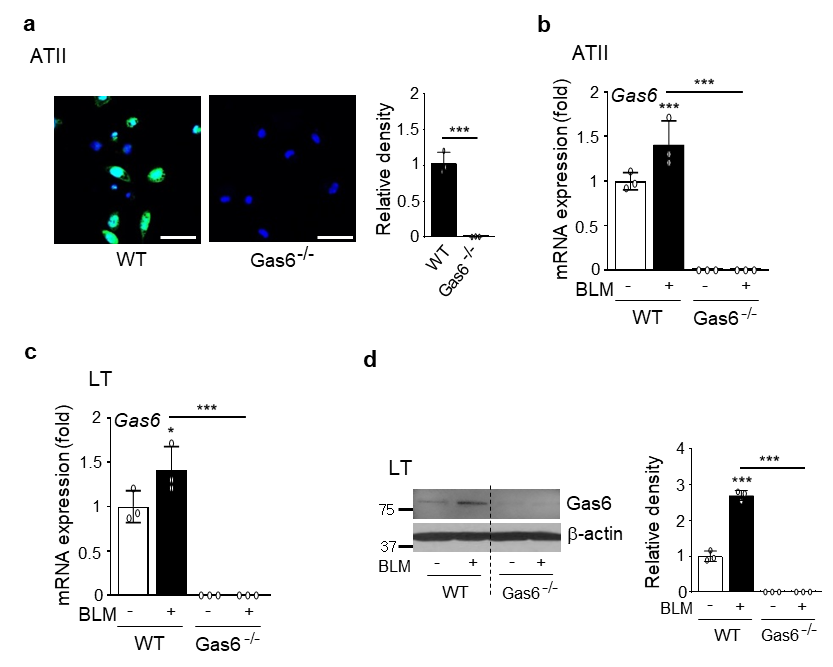
**

**Supplementary Fig. 5. Gas6 expression in ATII cells and lung tissue in WT and Gas6^−/−^ mice.** (**a**) Left: immunofluorescence staining for Gas6 (green) in primary ATII cells from WT and GAS6^−/−^ mice. Right: quantification of Gas6 staining. Original magnification: 400×. Scale bars: 20 μm. Imaging medium: Vectashield fluorescent mounting medium containing DAPI. (**b**) qRT-PCR of Gas6 in ATII cell samples. (**c**) Immunoblot analysis of Gas6 in lung tissue from WT and Gas6^−/−^ mice 14 days after BLM treatment. Right: Densitometric analysis of each band normalized to that of β-actin. (**d**) qRT-PCR of Gas6 in lung tissue. ***P < 0.001 compared with saline control or for WT vs. Gas6^−/−^ mice. Data were obtained from three replicates per condition with cells pooled from two mice per replicate (**a** *right*, **b**) or from three mice in each group (**c** *right*, **d**). The data are shown as the means ± SEM.


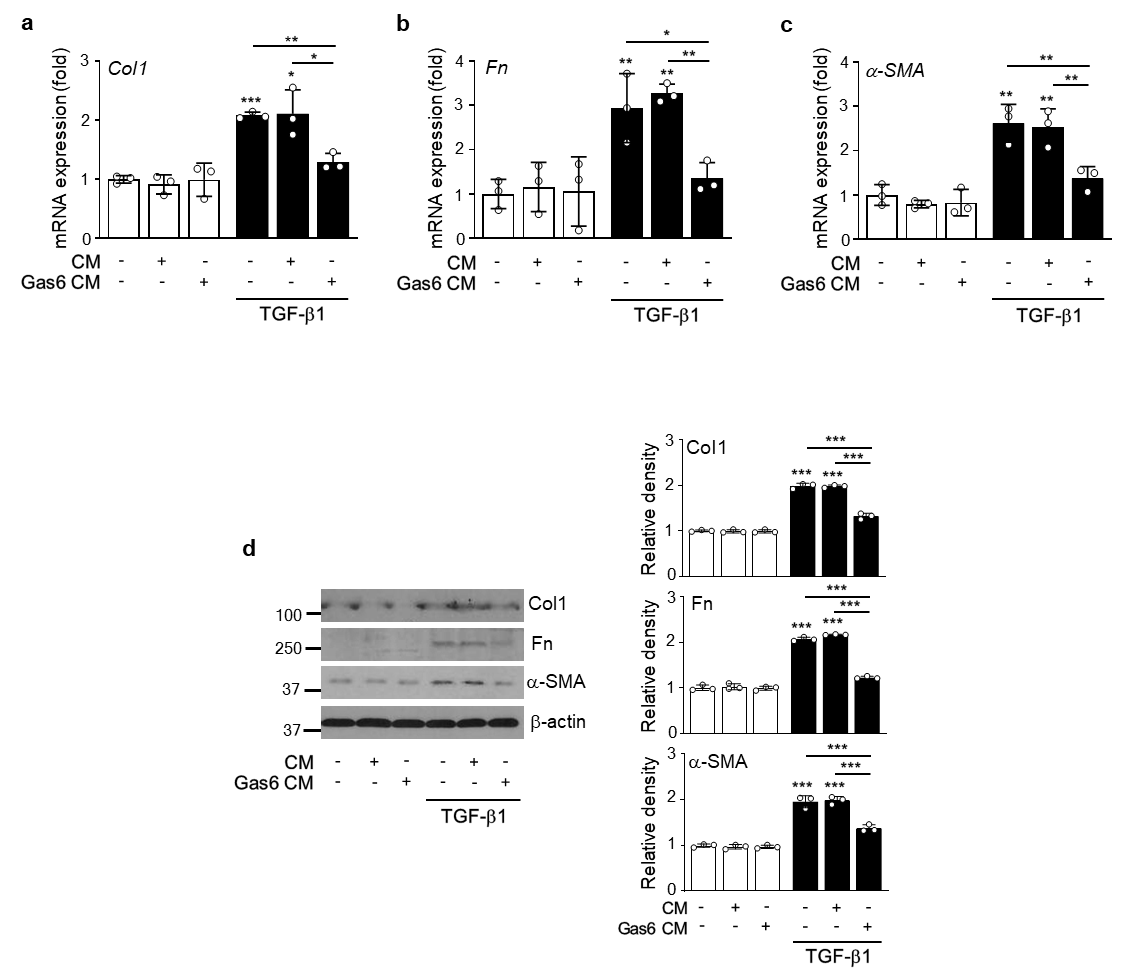


**Supplementary Fig. 6. Conditioned medium from LA-4 cells exposed to rGas6 reduces myofibroblast phenotypic marker in lung fibroblasts.** LA-4 cells were stimulated with rGas6 (400 ng/ml) for 20 h. Conditioned medium (CM) was added to MLg cells in the absence or presence of 10 ng/ml TGF-β1 for 24 h. (**a-c**) qRT-PCR of collagen type 1, fibronectin, and α-SMA in MLg cell samples. (**d**) Immunoblots of the indicated proteins in MLg cell lysates. Below: Densitometric analysis of each band normalized to that of β-actin. Values represent the means ± S.E.M. of three independent experiments. *P < 0.05, **P < 0.01, ***P < 0.001 compared with control.

**Table S1. List of antibodies**


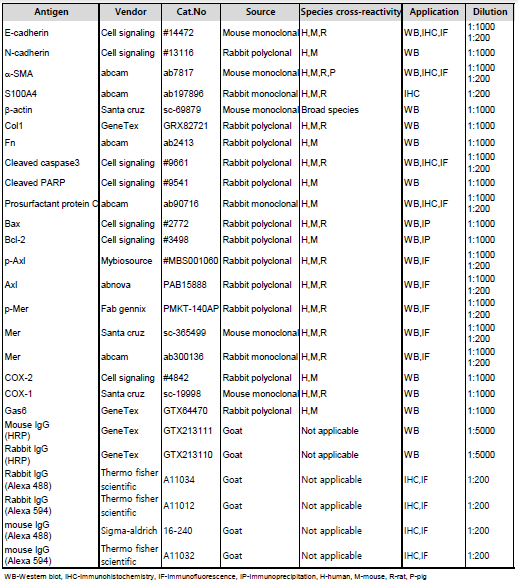


**Table S2. Primer sequences**


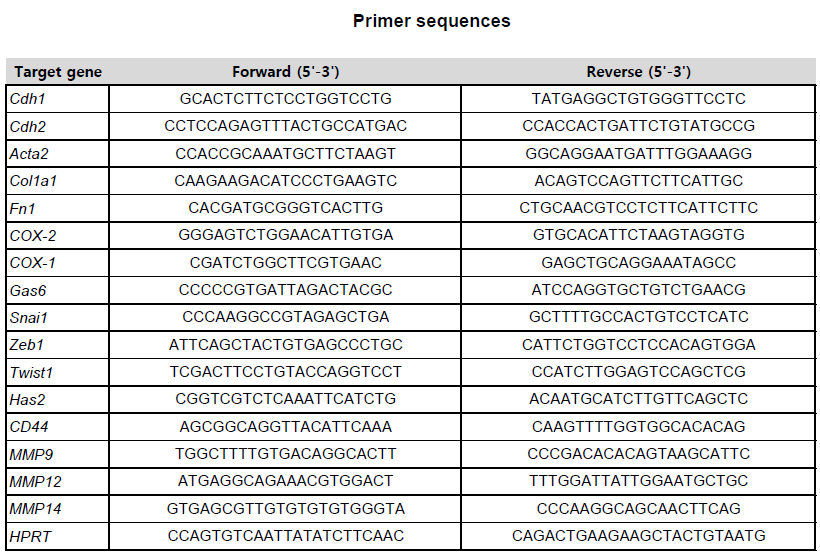

Supplement: Supplementary file 1 — Supplementary file1 (DOCX 925 KB) [file 10565_2024_9858_MOESM1_ESM.docx]
